# Monoallelic mutations in MMD2 cause autosomal dominant aggressive periodontitis

**DOI:** 10.1084/jem.20231911

**Published:** 2025-07-15

**Authors:** Tomoyuki Iwata, Yoko Mizoguchi, Tetsuya Yoshimoto, Miyuki Tsumura, Fumiaki Sakura, Jeffrey R. Johnson, Shinji Matsuda, Kazuhisa Ouhara, Yukiko Nagatani, Takaki Asano, Hidenori Ohnishi, Zenichiro Kato, Keichiro Mihara, Hirokazu Kanegane, Tomoya Ueda, Shinya Sasaki, Yuri Taniguchi, Yurika Ninomiya, Yoshinori Ohno, Kyoko Suzuki-Takedachi, Yusuke Sotomaru, Tetsushi Sakuma, Takashi Yamamoto, Yukiko Matsuda, Kodai Kume, Terukazu Sanui, Fusanori Nishimura, Mikihito Kajiya, Yasuyoshi Ueki, Hidemi Kurihara, Hiroyuki Morino, Satoshi Okada, Hideshi Kawakami, Noriyoshi Mizuno

**Affiliations:** 1 Department of Periodontal Medicine, Graduate School of Biomedical and Health Sciences, Hiroshima University, Hiroshima, Japan; 2 Department of Pediatrics, Graduate School of Biomedical and Health Sciences, Hiroshima University, Hiroshima, Japan; 3 Department of Innovation and Precision Dentistry, Hiroshima University Hospital, Hiroshima, Japan; 4 Department of Microbiology, Icahn School of Medicine at Mount Sinai, New York, NY, USA; 5 Global Health and Emerging Pathogens Institute, Icahn School of Medicine at Mount Sinai, New York, NY, USA; 6 Department of Dental Hygiene, University of Shizuoka Junior College, Shizuoka, Japan; 7 Department of Pediatrics, Gifu University Graduate School of Medicine, Gifu City, Japan; 8 Structural Medicine, United Graduate School of Drug Discovery and Medical Information Sciences, Gifu University, Gifu City, Japan; 9 International Regenerative Medical Center, Fujita Health University, Toyoake, Japan; 10 Department of Pediatrics and Developmental Biology, Graduate School of Medical and Dental Sciences, Tokyo Medical and Dental University, Tokyo, Japan; 11 Department of Biochemistry, Faculty of Medicine, Fukuoka University, Fukuoka, Japan; 12 Research Institute for Radiation Biology and Medicine, Hiroshima University, Hiroshima, Japan; 13 Natural Science Center for Basic Research and Development, Hiroshima University, Hiroshima, Japan; 14 Department of Mathematical and Life Sciences, Graduate School of Science, Hiroshima University, Higashi-Hiroshima, Japan; 15 Department of Epidemiology, Research Institute for Radiation Biology and Medicine, Hiroshima University, Hiroshima, Japan; 16 Division of Oral Rehabilitation, Department of Periodontology, Faculty of Dental Science, Kyushu University, Fukuoka, Japan; 17 Department of Biomedical Sciences and Comprehensive Care, Indiana University School of Dentistry, Indianapolis, USA; 18 Department of Medical Genetics, Tokushima University Graduate School of Biomedical Sciences, Tokushima, Japan

## Abstract

Aggressive periodontitis causes rapid destruction of periodontal tissue. It occurs at a young age with familial clustering. We report on the first time on molecular and cellular basis of a Mendelian form of autosomal dominant aggressive periodontitis. Monoallelic mutations in the monocyte to macrophage differentiation-associated 2 (*MMD2*) gene, encoding MMD2, in two Japanese families with autosomal dominant aggressive periodontitis are identified. Mutations, c.347 C>T (p.A116V) and c.377 G>C (p.R126P) in *MMD2*, disturbed fMLP-induced activation of Ras/ERK signaling. Additionally, abnormalities in the proteins of Golgi apparatus, a crucial contributor to innate immune signaling pathways, were identified in patients’ neutrophils. The knock-in and knockout mice exhibited alveolar bone loss by ligature-induced periodontitis, along with impaired fMLP-induced chemotaxis, as found in the patients with *MMD2* mutation. Our studies revealed that monoallelic mutations in *MMD2* underlie the impairment of neutrophil chemotaxis, which leads to the development of autosomal dominant aggressive periodontitis.

## Teaser

Monoallelic mutations, p.A116V and p.R126P, in MMD2 disturb fMLP-induced activation of Ras/ERK signaling and underlie the impairment of neutrophil chemotaxis, which leads to the development of the autosomal dominant form of aggressive periodontitis.

## Introduction

Periodontitis is a chronic inflammatory disease of the periodontal tissues caused by periodontopathic bacteria, leading to the destruction of periodontal tissues, including alveolar bone resorption. Aggressive periodontitis, formerly called early onset periodontitis or juvenile periodontitis, is a rare form of inflammatory periodontal disease that predominantly affects otherwise healthy subjects under 30 years of age and is characterized by familial clustering (Fine et al., 2018; Llorente and Griffiths, 2006; Nishimura et al., 1990; Trevilatto et al., 2002). The prevalence is 0.1–0.2% in Caucasians, 0.4–1.0% in Asians, and 1.0–3.0% in African-Americans (Susin et al., 2014). Patients with aggressive periodontitis experience rapid periodontal tissue destruction, resulting in the loss of multiple teeth, often without significant symptoms in other tissues. Despite its clinical significance, the genetic etiology of aggressive periodontitis remains poorly understood. The knowledge gap hinders early and accurate diagnosis, complicates the formulation of effective treatment strategies, and limits the development of targeted therapeutic interventions for this condition.

Neutrophil abnormalities are associated with the development of aggressive periodontitis. Impaired neutrophil chemotaxis and superoxide production have been reported in patients with aggressive periodontitis (Shapira et al., 1991; Van Dyke et al., 1980; Van Dyke et al., 1985). Moreover, severe periodontitis has been observed in patients with monogenic disorders that impair neutrophil numbers or function, such as severe congenital neutropenia (SCN) (Ye et al., 2011), cyclic neutropenia (Rezaei et al., 2008; Rezaei et al., 2007; Weston et al., 1991), Cohen’s syndrome (Alaluusua et al., 1997; Alipour et al., 2020), chronic granulomatous disease (CGD) (Giannopoulou et al., 2008), Papillon–Lefèvre syndrome (PLS) (Dalgic et al., 2011; Bullón et al., 2018; Toomes et al., 1999), Chédiak–Higashi syndrome (CHS) (Bailleul-Forestier et al., 2008; Delcourt-Debruyne et al., 2000; Thumbigere Math et al., 2018), and leukocyte adhesion deficiency (LAD) (Hajishengallis and Moutsopoulos, 2016; Dababneh et al., 2008). SCN and cyclic neutropenia result from impaired neutrophil granulocyte maturation, leading to a decreased neutrophil numbers. Chronic or intermittent neutropenia has also been reported in Cohen’s syndrome, and the patients with Cohen syndrome develop mild neutropenia (Duplomb et al., 2019; Fryns et al., 1996). In addition, the dysfunction of neutrophils due to Cohen syndrome, involving their increased adhesiveness, has also contributed to the exacerbation of periodontitis (Duplomb et al., 2014; Olivieri et al., 1998). Neutrophils of patients with CGD have impaired function, with an impaired oxidative burst following stimulation (Anjani et al., 2020). Among neutrophil dysfunctions, impaired chemotaxis can also cause severe periodontitis. PLS leads to a locally destructive inflammatory cycle due to impaired neutrophil chemotactic ability and continuous recruitment and accumulation of hyperactive/reactive neutrophils. Neutrophils of patients with CHS have impaired chemotaxis and bacterial killing associated with a lack of cathepsin G and elastase in their granules. LAD is characterized by leukocytes’ inability to migrate to infection sites and eliminate offending microbes. However, periodontitis in LAD-I is not solely an infection but rather a microbe-triggered inflammatory disease (Moutsopoulos et al., 2015; Moutsopoulos et al., 2014). LPSs have been detected within inflammatory lesions of LAD-I periodontitis and in proximity to inflammatory cells, suggesting microbial triggering of inflammatory responses (Moutsopoulos et al., 2015). Consequently, periodontal inflammation and bone loss are hypothesized to result from the absence of functional tissue neutrophils due to severe reductions in numbers, defective recruitment, impaired transmigration, or impaired bacterial killing.

We here identified monoallelic mutations, p.A116V and p.R126P, in the monocyte to macrophage differentiation-associated 2 (MMD2) gene, which encodes MMD2, in two unrelated families with aggressive periodontitis. Neutrophils from the patients exhibited abnormalities in chemotaxis. Abnormalities of Golgi apparatus, where MMD2 express, and altered fMLP-induced ERK signaling identified in the patients’ neutrophil were suspected to underlie the impaired chemotaxis. Knock-in mice with equivalent MMD2 variants to those found in patients developed ligation-induced periodontitis and recapitulated the symptoms. These observations support the concept of the new Mendelian disease causing aggressive periodontitis linked to monoallelic mutations in *MMD2*.

## Results and discussion

### Families with aggressive periodontitis

**Figure 1.**
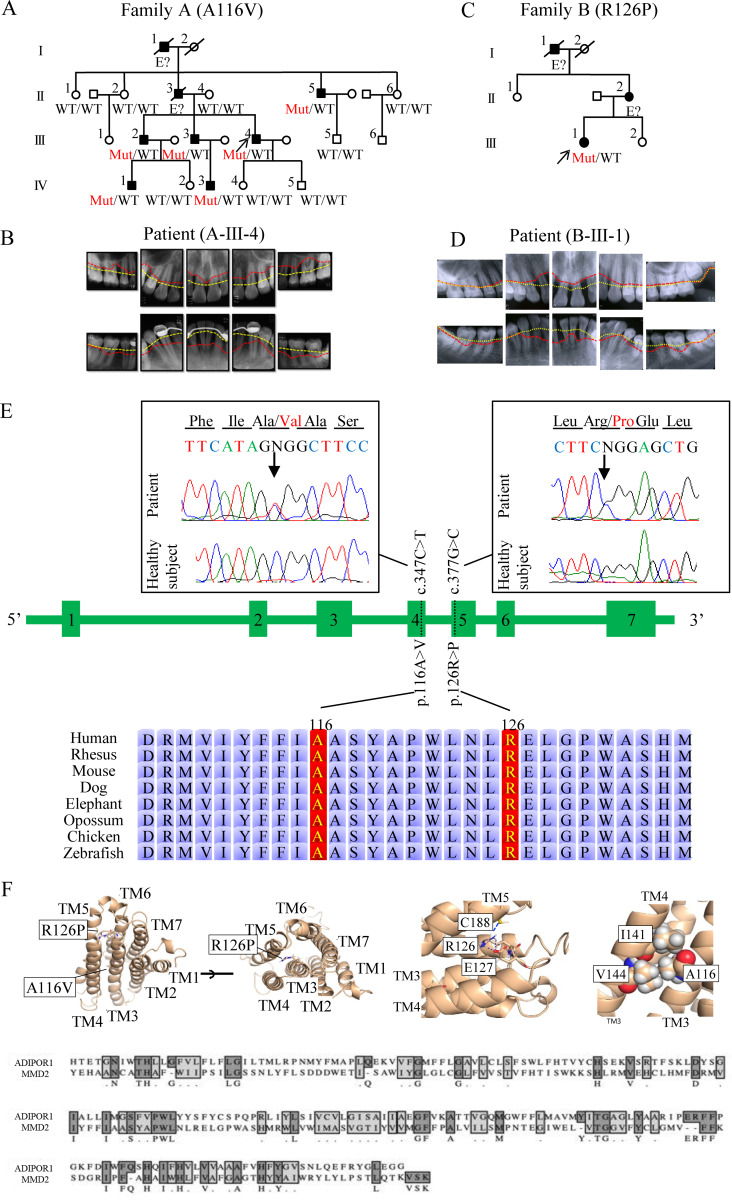
**Characteristic findings in patients with *MMD2* mutations and heterozygous *MMD2* mutations identified in two families. (A)** The families’ pedigrees with p.A116V mutation. Arrows indicate the proband. Filled and open symbols represent affected and unaffected subjects, respectively. Genotypes of the variants c.347C>T and c.377G>C are shown under the number of samples. **(B)** X-ray images of the 26-year-old patient A-III-4. The normal alveolar bone line is indicated by a yellow dotted line, while the patient’s alveolar bone line is indicated by a red dotted line. **(C)** The families’ pedigrees with p.R126P mutation. **(D)** X-ray images of the 16-year-old patient B-III-1. **(E)** The intron/exon organization and amino acid alignments. Sanger sequencing of *MMD2* gene exons 4 and 5 with or without the mutation. The amino acid sequences that were completely conserved among vertebrates. **(F)** Three-dimensional structural representations of MMD2.

**Figure S1.**
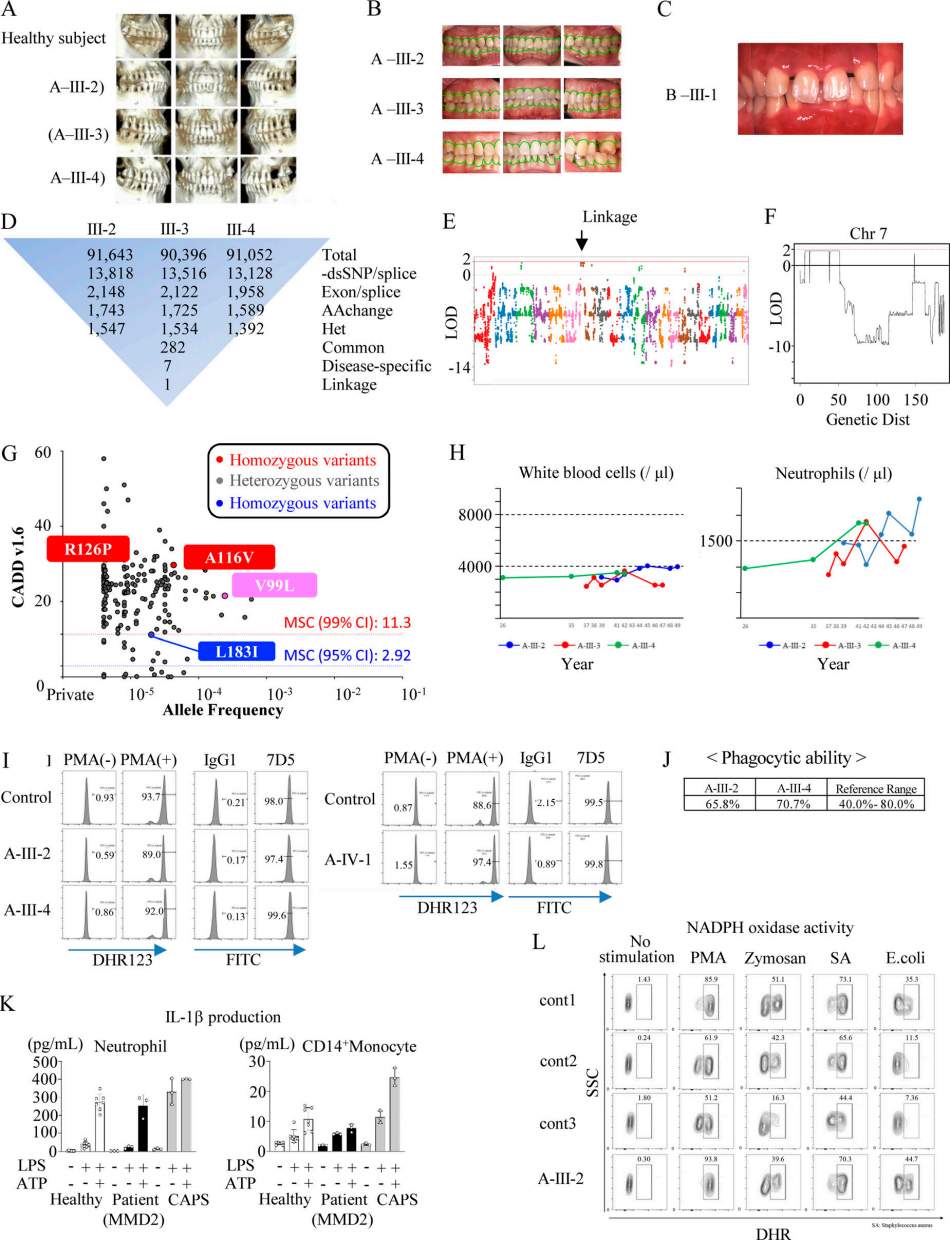
**Clinical photographs, population genetics, and cellular analyses in patients with *MMD2* mutations. (A)** Computed tomography images of age-matched healthy subjects, the 45-year-old patient A-III-2, the 44-year-old patient A-III-3, and the 40-year-old patient A-III-4. **(B)** Clinical photographs of family A. The normal gingival line is indicated by a green line. **(C)** Clinical photographs of the 16-year-old B-III-1. The gums are reddened. **(D)** Filtering and numbers of called variants. **(E and F)** Linkage analysis of the studied family A. Arrows indicate the position of the *MMD2* gene. **(G)** CADD score (*y* axis) versus allele frequency (*x* axis) for all the variants of MMD2 found in gnomeAD. **(H)** Temporal changes in white blood cell count and temporal changes in neutrophils. **(I)** Superoxide production ability as determined using DHR123 antibody. Diagnosis of chronic granulomatosis using 7D5 antibody. **(J)** Phagocytic ability. **(K)** IL-1β production in neutrophils and monocytes of a patient (A-III-2, A-III-4, and A-IV-1). **(L)** FACS histogram showing superoxide production in neutrophils of a patient (A-III-2) and healthy subjects with or without PMA, zymosan, *S*.* aureus* (SA), and *E. coli*. Activity was assessed using DHR. DHR, dihydrorhodamine.

We investigated two Japanese families with dominantly inherited aggressive periodontitis. In family A, the proband (A-III-4) was admitted to our hospital at the age of 24 with complaints of gingival swelling and pain since his late teens. The upper left molar was non-salvageable and required extraction, with deep periodontal pockets also noted in several other teeth. The proband’s two brothers (A-III-2 and A-III-3) exhibited similar symptoms in their late teens. The mean periodontal pocket depths (>3 mm is considered alveolar bone resorption) were 4.5, 3.9, and 4.77 mm for A-III-2, A-III-3, and A-III-4, at ages 30, 27, and 24, respectively. Family history revealed that the proband’s deceased grandfather (A-I-1), father (A-II-3), and uncle (A-II-5) also suffered from aggressive periodontitis, with both A-I-1 and A-II-3 requiring dentures from a young age. A-IV-1, aged 18, and A-IV-3, aged 17, exhibited alveolar bone loss, even though they are young (Fig. 1 A). Interestingly, the patients were systemically healthy, except that they had severe periodontitis with alveolar bone loss. Computed tomography images of A-III-2, A-III-3, and A-III-4 at ages 45, 44, and 40, respectively, and X-ray images of A-III-4 at age 24 demonstrated a damaged tooth exhibiting half to one-third alveolar bone resorption around the tooth root (Fig. 1 B and Fig. S1 A) compared with that of a healthy subject, even though the patients had received professional dental treatment from their teenage years to their 40s. Intraoral photographs further displayed exposed tooth roots as a result of gingival recession (Fig. S1 B).

In family B, the proband (B-III-1) was admitted to our hospital at the age of 16 with complaints of gingival redness, swelling, and pain (Fig. S1 C). Clinical examination revealed the presence of deep periodontal pockets. The mean periodontal pocket depth was 3.64 mm. The proband’s deceased grandfather (B-I-1) and mother (B-II-2) also suffered from aggressive periodontitis, with B-I-1 requiring dentures from a young age (Fig. 1 C). X-ray images of B-III-1 at age 16 demonstrated a damaged tooth exhibiting half to one-third alveolar bone resorption around the root (Fig. 1 D).

### Identification of the MMD2 mutation

We performed whole-exome sequencing (WES) using genomic DNA (gDNA) from the three affected subjects with aggressive periodontitis (A-III-2, A-III-3, and A-III-4). WES identified seven possible heterozygous candidate variants, which were shared among three affected subjects (Fig. S1 D). We also performed linkage analysis, which detected two regions with logarithm of odds scores of 1.8047 and 1.8058 in chromosomes 3 and 7, respectively (Fig. S1, E and F). Among seven variants identified in WES, the variant c.347 C>T (p.A116V) in *MMD2* was the only variant located in the linked region. The nucleotide and amino acid sequences of the mutated region were shown to be highly conserved among vertebrates (Fig. 1 E). The p.A116V *MMD2* variant was absent in 275 healthy Japanese subjects. Subsequently, Sanger sequencing of all exons of 102 Japanese aggressive periodontitis patients revealed a patient with the p.R126P variant (B-III-1). Although the protein structure of MMD2 has not yet been determined, it is predicted to possess a seven-transmembrane structure based on its homology with adiponectin receptor 1. Two variants (p.A116V and p.R126P) are located in TM3. Given that R126 is expected to interact with neighboring residues, E127 and C188, the loss of the arginine side chain may lead to a reduction in the stability of this helix bundle. Additionally, as A116 forms a hydrophobic core with residues from opposing helices, such as A141 and V144, the presence of V116 may potentially result in steric hindrance (Fig. 1 F). Based on these findings, the p.A116V and p.R126P *MMD2* variants identified in the current study were rare and were suspected to be pathogenic and causative of aggressive periodontitis. Along with the results of WES, the absence of CGD-related variants in *NCF1*, *NCF2*, *NCF4*, *CYBB*, *CYBA*, *CYBC1*, and *ITGB2*, in addition to known responsible genes in congenital neutropenia, was confirmed by long-read sequencing. The CADD scores and allele frequencies of all MMD2 variants in gnomAD revealed relatively higher CADD scores for the p.A116V and p.R126P mutations (Fig. S1 G).

### Characteristics of the patients’ peripheral blood and bone marrow

The peripheral blood obtained from affected subjects (A-III-2, A-III-3, and A-III-4) were studied. The white blood cell count was mildly decreased in all patients analyzed; however, the ratio of neutrophils to all leukocytes remained normal. The absolute number of neutrophils was close to the lower limit of the normal range or mildly decreased (Fig. S1 H). The normal oxidative burst activity and expression of gp91^*phox*^ (7D5) were confirmed in A-III-2, A-III-4, and A-IV-1, ruling out the possibility of classic CGD (Fig. S1 I). Additionally, neutrophils from A-III-2 and A-III-4 displayed normal phagocytic ability (Fig. S1 J). The secretion of IL-1β in neutrophils and monocytes from A-III-2, A-III-4, and A-IV-1 after stimulation with LPS and ATP was comparable with that of healthy subjects (Fig. S1 K). The ability of A-III-2 to produce ROS was shown to be normal (Fig. S1 L). Lymphocyte subset analysis was performed in A-III-2 and A-III-4 by multicolor flow cytometry (Fig. S3 F). Although a decreased frequency of CD19^+^ B cells was observed in both patients, serum immunoglobulin levels remained within normal ranges. The IgE levels in A-III-2, A-III-4, and A-IV-1 were within normal limits (A-III-2: 30 IU/ml, A-III-4: 64.3 IU/ml, and A-IV-1: <20 IU/ml) (Fig. S3 G). Moreover, the IgG levels in A-III-2 was within normal limits (1,120 mg/dl).

**Figure S2.**
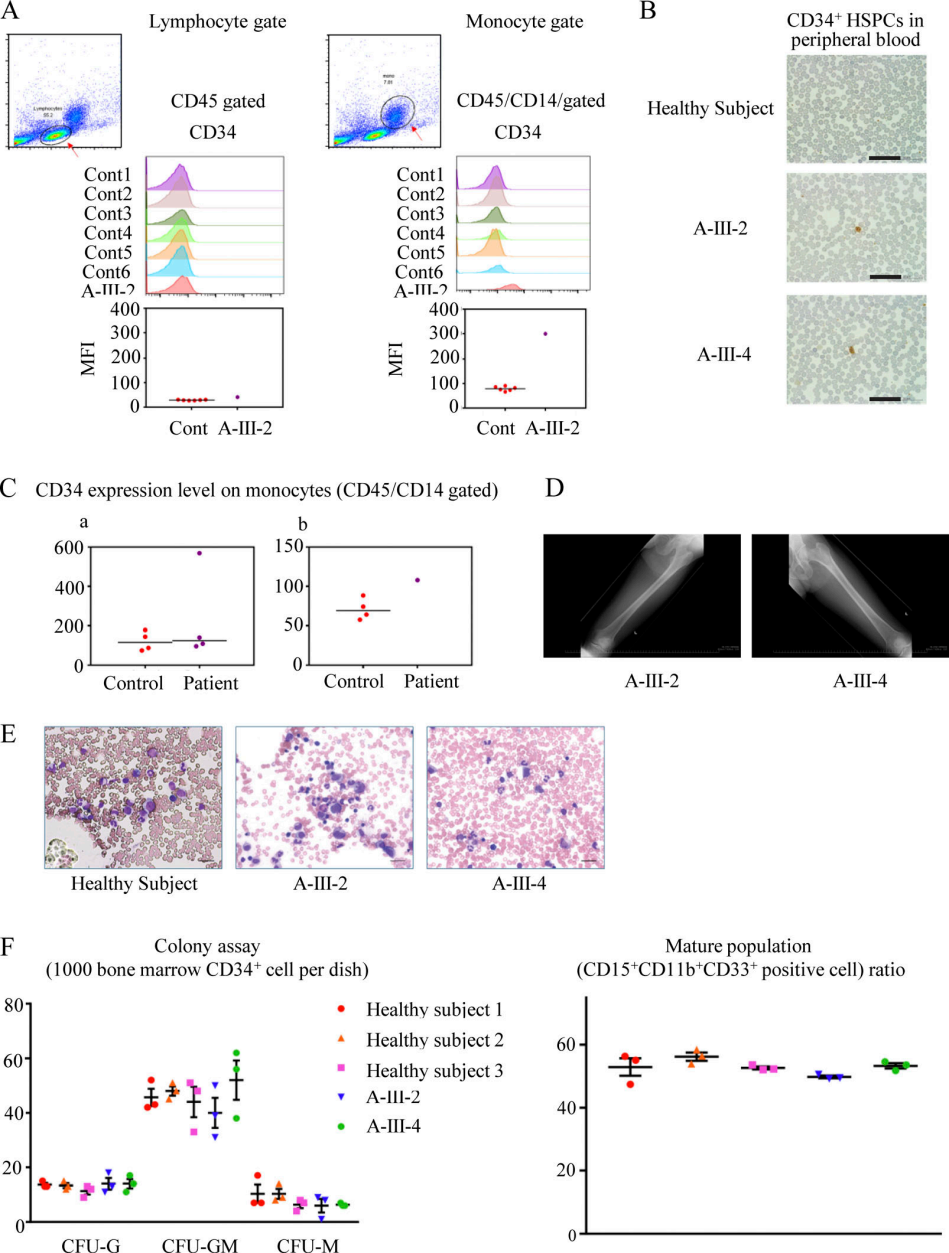
**Characteristics of the patients’ peripheral blood and bone marrow. (A)** The percentage of CD34^+^ cells were assessed using peripheral blood. The cells were gated from each lymphocytes (low SSC and CD45^+^) or monocytes (medium SSC with CD45^+^/CD14^+^) and analyzed by flow cytometry. The upper figure shows the histogram of CD34 expression for each subject, with lymphocytes on the right and monocytes on the left. The lower figure summarized the data including the mean fluorescent intensities (MFI) of CD34 expression collected from a patient (A-Ⅲ-2) and six healthy controls for each gating strategy. MFI of CD34 expression in monocytes was assessed in five subjects with *Mmd2*^A116V/+^. **(a)** The data from patients (A-Ⅲ-2, A-Ⅲ-3, A-Ⅳ-1, and A-Ⅳ-2) and healthy controls were displayed. **(b)** The data from patient (A-Ⅲ-4) was shown with healthy controls. The results of two independent experiments were shown. **(B)** Identification of CD34^+^ cells in peripheral blood using immunohistochemical staining. CD34^+^ cells in peripheral blood of patients III-2 and III-4 are shown. Scale bar = 50 μm. **(C)** X rays of femur in patients A-III-2 and A-III-4. **(D)** CD34 expression level on monocytes (CD45/CD14 gated). **(E)** Images of the bone marrow cells of healthy subjects and patients A-III-2 and A-III-4. Scale bar = 25 μm. **(F)** The induced colony number (CFU-G, CFU-granulocyte; CFU-GM, CFU-granulocyte/macrophage; CFU-M, CFU-macrophage) and mature population (CD15^+^CD11b^+^CD33^+^ cells) of bone marrow cells are shown.

Interestingly, an increase in CD34^+^ hematopoietic stem and progenitor cells (HSPCs) within the CD45^+^/CD14^+^ cell population was noted in patients A-III-2 and A-III-4 (Fig. S2 A). This increase in CD34^+^ HSPCs was persistently observed in peripheral blood of A-III-2 and A-III-4, confirmed by immunohistochemistry (Fig. S2 B). On the other hand, increase in CD34^+^ HSPCs was not found in A-III-3, A-IV-1, A-IV-2 and the six control subjects (Fig. S2 C). Due to the lack of consent, we could not investigate HSPCs in peripheral blood from a patient with p.R126P mutation. Generally, only a small population of HSPCs is present in the circulation under steady-state conditions (de Kruijf et al., 2020). This elevation is typically observed transiently in peripheral blood following high-dose chemotherapy, mobilization with granulocyte CSF (G-CSF), or in patients with osteopetrosis. X-ray photographs of the femurs of A-III-2 and A-III-4 showed no signs of osteopetrosis (Fig. S2 D).

The bone marrow aspiration from A-III-2 and A-III-4 showed normal findings (Fig. S2 E). The granulocyte differentiation experiments using the patients’ CD34^+^ HSPCs showed normal granulopoiesis without the finding of maturation arrest, which is a characteristic finding in patients with SCN (Fig. S2 F). Although A-III-2 and A-III-4 showed increased CD34^+^ HSPCs in the peripheral blood, no clear hematopoietic abnormalities were observed in the bone marrow of either, suggesting that these cells may have leaked from the bone marrow to the peripheral blood. Further accumulation and examination of cases are necessary to clarify the association between increased CD34^+^ HSPCs in the peripheral blood and monoallelic mutations in *MMD2*.

### MMD2 mutation knock-in mice

**Figure S3.**
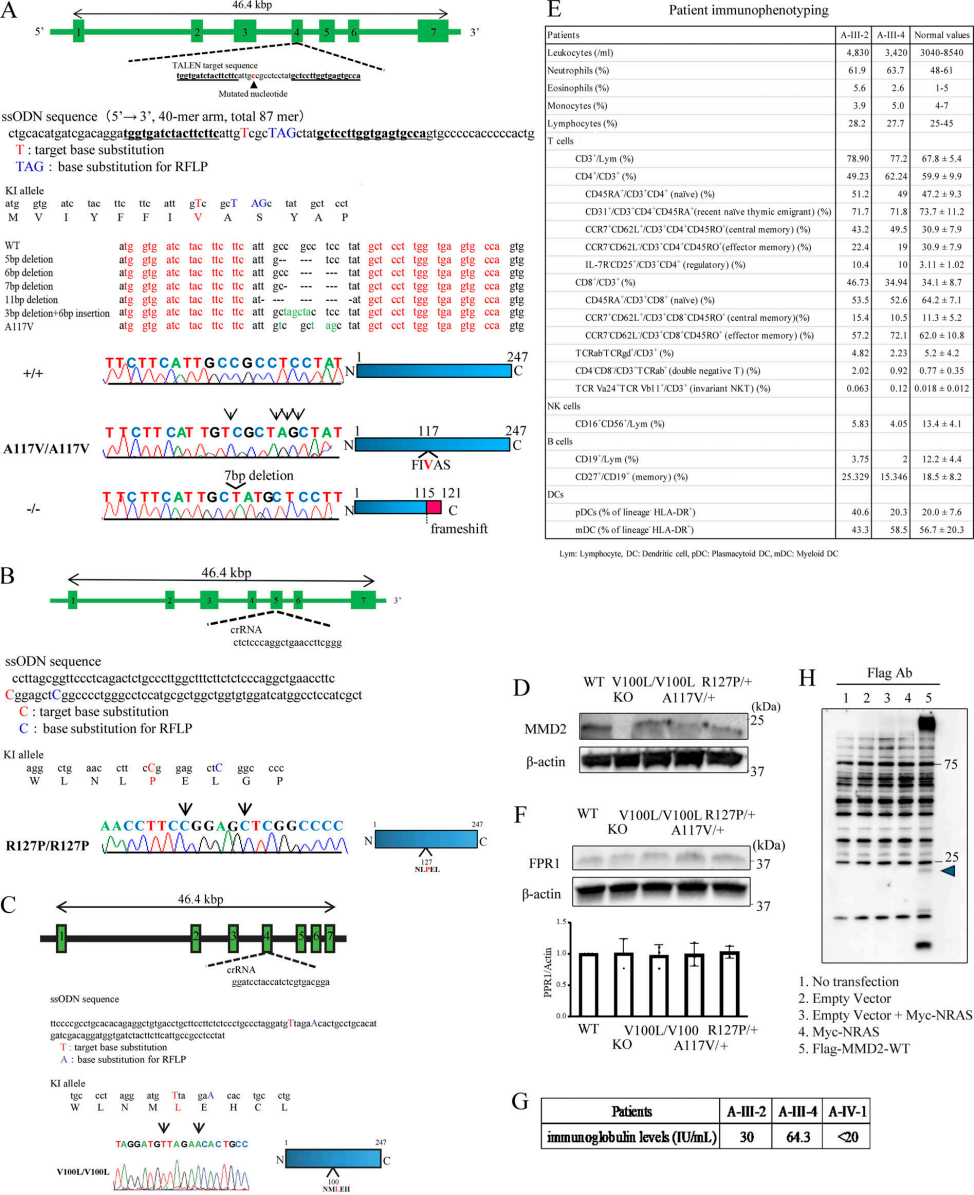
**Generation of *Mmd2* A117V, R127P, and V100L knock-in mice using platinum TALEN and CRISPR-Cas9 and immunophenotyping of patients with p.A116V mutation. (A)** The genomic structure of *Mmd2*, indicating the two binding sites of the TALENs, is shown. TALEN pairs were designed to bind exon 4 of the *Mmd2* gene. The sequence information of *Mmd2* mutant alleles, specifically, sequences obtained from mutant mice, which were generated by microinjection of TALEN mRNA. The DNA sequences that were used for designing the TALENs are highlighted in red. Nucleotide mutations and indels are shown. The reference nucleotide C was substituted with variant nucleotide T in the mutant sample. A 7-bp deletion resulted in a frameshift and thus in truncated proteins. **(B)** crRNA was designed to bind exon 5 of the *Mmd2* gene. **(C)** crRNA was designed to bind exon 4 of the *Mmd2* gene. **(D)** MMD2 protein expression in *Mmd2*^+/+^, *Mmd2*^−/−^, *Mmd2*^V100L/V100L^, *Mmd2*^A117V/A117V^, and *Mmd2*^R127P/R127P^ mice neutrophils. **(E)** FPR1 protein expression in *Mmd2*^+/+^, *Mmd2*^−/−^, *Mmd2*^V100L/V100L^, *Mmd2*^A117V/+^, and *Mmd2*^R127P/+^ mice neutrophils. **(F)** Patient immunophenotyping. **(G)** Patient IgE levels. **(H)** Immunoblots were performed using HEK293T cells without transfection (lane 1, no transfection) and HEK293T cells transfected with the empty vector (lane 2), the empty vector and Myc-NRAS vector (Lane 3), the Myc-NRAS vector (lane 4), and Flag-MMD2-WT vector (Lane 5). Source data are available for this figure: SourceData FS3.

To characterize the functional significance of p.A116V, p.R126P, and p.V99L mutation in *MMD2*, we generated a knock-in mouse model (*Mmd2*^A117V/A117V^, *Mmd2*^R127P/R127P^, and *Mmd2*^V100L/V100L^ mice) carrying an amino acid substitution in *Mmd2*, which corresponded to the p.A116V, p.R126P, and p.V99L mutations in the human *MMD2* gene. The p.V99L is a nonpathogenic variant that is also found in healthy subjects. Platinum TALENs were designed to generate a double-stranded break near the targeted nucleotide c.350C>T (p.A117V) in exon 4 of the *Mmd2* gene. The oligonucleotide contained four single-nucleotide differences, including the nonsynonymous C>T substitution encoding the mouse p.A117V mutation and a synonymous change that introduced a PstI site. Genome editing of the targeted nucleotide mutations resulted in five random deletions and one indel, as the 5-, 7-, and 11-bp deletions produced frameshift mutations. We used the mice with 7-bp deletions in *Mmd2* as knock-out mice (*Mmd2*^−/−^ mice; Fig. S3 A). CRISPR-Cas9 was designed in proximity to the targeted nucleotide c.380G>C (p.R127P) in exon 5 of the *Mmd2* gene. The oligonucleotide included two single-nucleotide variations, including the nonsynonymous G>C substitution encoding the mouse p.R127P mutation and a synonymous change that unrecognized an ApaI recognition site (Fig. S3 B). Similarly, CRISPR-Cas9 was designed in proximity to the targeted nucleotide c.298G>T (p.V100L) in exon 4 of the *Mmd2* gene. The oligonucleotide contained two single-nucleotide differences, including the nonsynonymous G>T substitution encoding the mouse p.V100L mutation and a synonymous change that unrecognized a Bsp1286I site (Fig. S3 C). Expression levels of MMD2 protein in neutrophils from WT (*Mmd2*^+/+^), *Mmd2*^V100L/V100L^, *Mmd2*^A117V/A117V^, and *Mmd2*^R127P/R127P^ mice was comparable (Fig. S3 D). There was no difference in the protein expression of FPR1, the receptor of fMPL, among the genetically modified mice (Fig. S3 E).

### Neutrophil from the patients impaired chemotaxis without altering MMD2 expression

**Figure 2.**
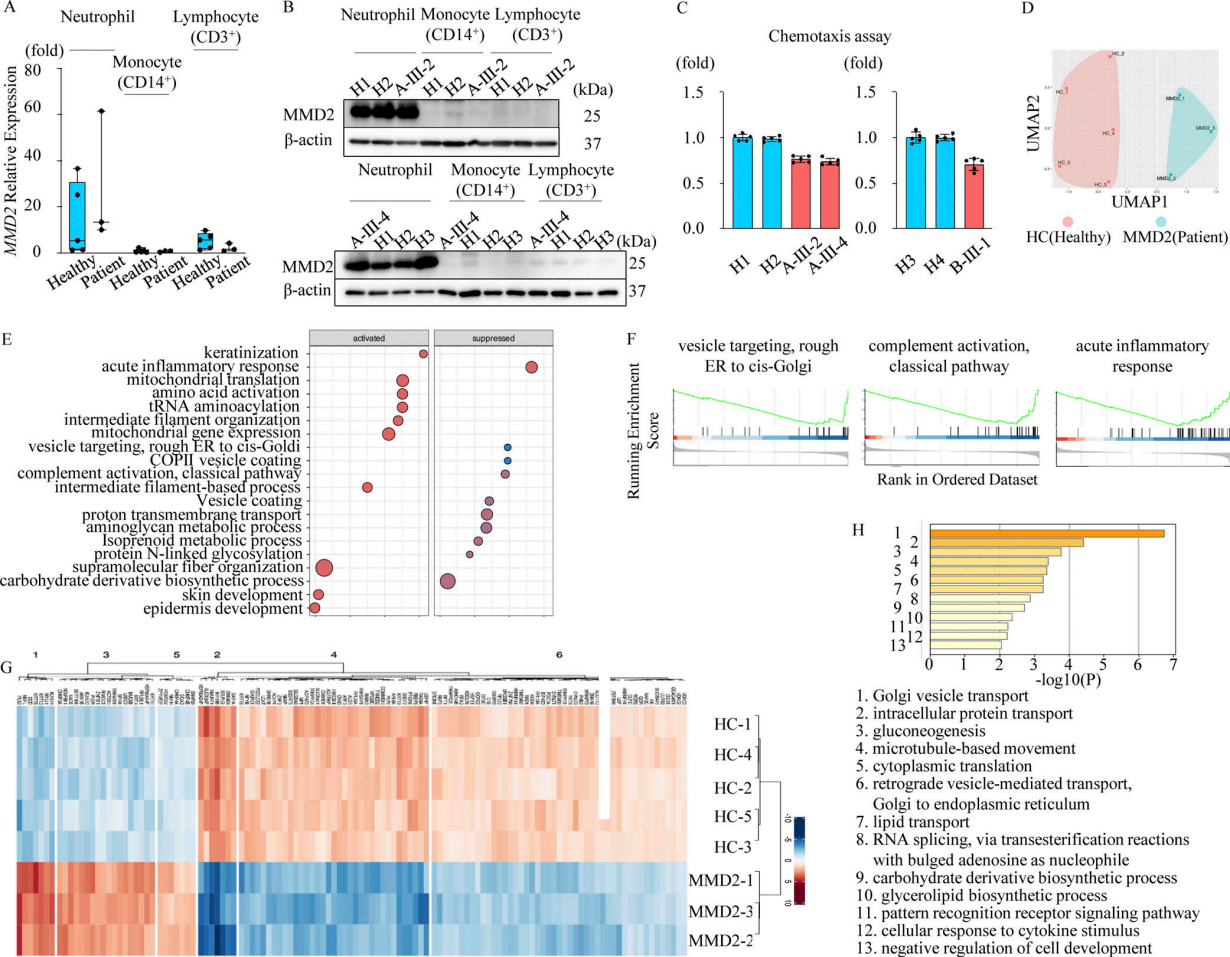
**Characteristics of neutrophils with *MMD2* mutations. (A)** *MMD2* expression, as assessed by real-time PCR on mRNA from patient’s (A-III-2 and A-III-4) and healthy subjects *n* = 3–5. **(B)** Immunoblotting analysis of MMD2 expression in neutrophils, monocytes, and lymphocytes from patient’s (A-III-2 and A-III-4) and healthy subjects. **(C)** Neutrophil chemotaxis induced by fMLP (100 nM, 4 h) was decreased in patients A-III-2, A-III-4, and B-III-1 compared with that in healthy subjects (H1, H2, H3, and H4) *n* = 5. **(D)** UMAP visualization in protein expression profiles of MMD2 patients (*n* = 3; blue) and healthy controls (*n* = 5; red). **(E)** Top-10 GO terms significantly upregulated or downregulated in MMD2 patients compared with healthy controls (P value <0.05). The color scale indicates the P value, and the size of the circle indicates the number of genes in the GO term. The Rene ratio indicates the number of core genes against the number of genes in the pathway. **(F)** GSEA plot of significantly suppressed GO terms. Proteins are ranked in decreasing order based on log 2 fold change of the differentially expression analysis. The color scale in the bottom graph shows upregulated (red) and downregulated (blue) expression in MMD2 patients. **(G)** Hierarchical clustering analysis based on differentially expressed proteins. The color scale shows log_2_ fold change comparing MMD2 patients with healthy controls, with red indicating upregulation and blue indicating downregulation. **(H)** GO analysis of downregulated proteins in MMD2 patients. The x axis shows the log_10_ P value of enriched GO terms. Source data are available for this figure: SourceData F2.

Neutrophils highly express *MMD2* mRNA and compared with monocytes and lymphocytes, and the expression level of *MMD2* mRNA in those cells did not alter between patients with the p.A116V mutation (A-III-2 and A-III-4) and healthy subjects (Fig. 2 A). In addition, the normal level of MMD2 protein in these cells was observed in A-III-2 and A-III-4 (Fig. 2 B). Severe periodontitis is observed in patients with monogenic disorders that impair neutrophil count or function. On the other hand, patients with monoallelic mutations in *MMD2* mutations do not exhibit severe neutropenia. Therefore, we focused on neutrophils, which have high MMD2 expression, to analyze chemotaxis. Neutrophil chemotaxis, evaluated through stimulation with fMLP, decreased in the patients (A-III-2, A-III-4, and B-III-1) upon comparison with the level of age-matched healthy subjects (Fig. 2 C).

### Proteomic analysis of patient’s neutrophil reveals Golgi apparatus abnormalities and innate immune abnormalities

To gain a deeper understanding of the underlying mechanisms contributing to impaired neutrophil chemotaxis in the context of abnormal MMD2 function, we conducted a mass spectrometry–based proteomic analysis of neutrophils from patients and healthy controls. The uniform manifold approximation and projection (UMAP) analysis definitively segregates the patients from healthy subjects (Fig. 2 D), proving that MMD2 dysfunction has a considerable impact on neutrophils. It is known that MMD2 is exclusively localized in the Golgi apparatus and belongs to the Golgi scaffold proteins (Jin et al., 2012; Peng et al., 2014). The gene set enrichment analysis (GSEA) results showed that proteins associated with the Golgi apparatus, as defined by gene ontology (GO) terms such as “vesicle targeting, rough ER to cis-Golgi,” and “COPII vesicle coating,” were downregulated in patients with monoallelic mutations in *MMD2* (Fig. 2 E). GSEA also indicated reduced expression of proteins linked to “complement activation” and “acute inflammatory response” in neutrophils of patients (Fig. 2 F).

We next conducted a differential expression analysis (DEA) to validate the GSEA results, comparing patients and healthy controls. The protein expression profiles of neutrophils harboring the p.A116V mutant MMD2 exhibited decreased expression of 89 proteins and increased expression of 32 proteins (Fig. 2 G). The GO analysis of downregulated proteins revealed that proteins related to the Golgi apparatus function and the pattern recognition receptor signaling pathway were significantly enriched, confirming the GSEA results (Fig. 2 H). These findings suggest that a monoallelic mutation, p.A116V, in *MMD2* affect Golgi apparatus function and significantly influence neutrophil function, which plays a crucial role in innate immunity.

### Impaired neutrophil chemotaxis and severe inflammatory osteopathic destruction in a mouse model

**Figure 3.**
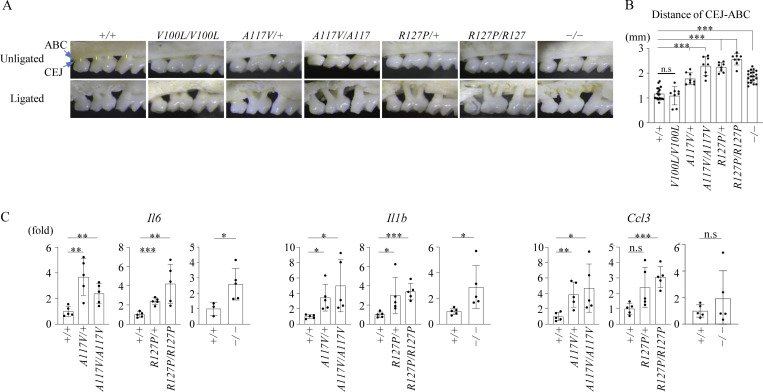
**Induction of periodontitis and mRNA levels of pro-inflammatory cytokines in gingival tissues. (A)** Representative photographs of the maxilla of treated and untreated mice. Scale bar = 1 mm. **(B)** Schematics of periodontal bone loss measurements. The distances between the cement–enamel junction and the alveolar bone crest (CEJ-ABC) at the distal facial side of the first molar, at the mesial and distal facial sides of the second molar, and at the mesial facial side of the third molar were measured (*Mmd2*^+/+^, *n* = 19; *Mmd2*^V100L/V100L^, *n* = 8; *Mmd2*^A117V/+^, *n* = 8; *Mmd2*^A117V/A117V^, *n* = 8; *Mmd2*^R127P/+^, *n* = 8; *Mmd2*^R127P/R127P^, *n* = 8; *Mmd2*^−/−^, *n* = 20). Measurements of the distances of CEJ-ABC were performed by personnel blinded to sample information. *Mmd2*^+/+^ versus *Mmd2*^V100L/V100L^; P = 0.9926, *Mmd2*^+/+^ versus *Mmd2*^A117V/+^; P < 0.0001, *Mmd2*^+/+^ versus *Mmd2*^A117V/A117V^; P < 0.0001, *Mmd2*^+/+^ versus *Mmd2*^R127P/+^; P < 0.0001, *Mmd2*^+/+^ versus *Mmd2*^R127P/R127P^; P < 0.0001, *Mmd2*^+/+^ versus *Mmd2*^−/−^; P < 0.0001. **(C)** qPCR analysis of inflammatory genes in the gingival tissues at 24 h after ligation. 18S was used for normalization. (*Mmd2*^+/+^, *n* = 5; *Mmd2*^A117V/+^, *n* = 5; *Mmd2*^A117V/A117V^, *n* = 5; *Mmd2*^R127P/+^, *n* = 5; *Mmd2*^R127P/R127P^, *n* = 5; *Mmd2*^−/−^, *n* = 5). *Il6* (*Mmd2*^+/+^ versus *Mmd2*^A117V/+^; P = 0.0037, *Mmd2*^+/+^ versus *Mmd2*^A117V/A117V^; P = 0.0080, *Mmd2*^+/+^ versus *Mmd2*^R127P/+^; P = 0.0001, *Mmd2*^+/+^ versus *Mmd2*^R127P/R127P^; P = 0.0080, *Mmd2*^+/+^ versus *Mmd2*^−/−^; P = 0.0469). *Il1b* (*Mmd2*^+/+^ versus *Mmd2*^A117V/+^; P = 0.0142, *Mmd2*^+/+^ versus *Mmd2*^A117V/A117V^; P = 0.0297, *Mmd2*^+/+^ versus *Mmd2*^R127P/+^; P = 0.0465, *Mmd2*^+/+^ versus *Mmd2*^R127P/R127P^; P = 0.0003, *Mmd2*^+/+^ versus *Mmd2*^−/−^; P = 0.0370). *Ccl3* (*Mmd2*^+/+^ versus *Mmd2*^A117V/+^; P = 0.0059, *Mmd2*^+/+^ versus *Mmd2*^A117V/A117V^; P = 0.0315, *Mmd2*^+/+^ versus *Mmd2*^R127P/+^; P = 0.0506, *Mmd2*^+/+^ versus *Mmd2*^R127P/R127P^; P = 0.0003, *Mmd2*^+/+^ versus *Mmd2*^−/−^; P = 0.0881). **(B and C)** Data are presented as mean ± SD. *P < 0.05, **P < 0.01, and ***P < 0.001 with one-way ANOVA with Tukey–Kramer test (B and C) or two-tailed unpaired *t* test (C, *Mmd2*^+/+^ versus *Mmd2*^−/−^). ns = not significant. Each dot represents a biologically independent mouse.

A ligation-induced periodontitis mouse model was used to examine whether Mmd2 mutations confer a genetic predisposition to severe periodontitis (Abe and Hajishengallis, 2013). *Mmd2*^A117V/+^, *Mmd2*^A117V/A117V^, *Mmd2*^R127P/+^, *Mmd2*^R127P/R127P^, and *Mmd2*^−/−^ mice in the ligature-induced periodontitis showed significantly increased alveolar bone loss compared with *Mmd2*^+/+^ and *Mmd2*^V100L/V100L^ mice (Fig. 3, A and B). Moreover, the mRNA levels of *Il6*, *Il1b*, and *Ccl3* in the gingival tissues of *Mmd2*^A117V/+^, *Mmd2*^A117V/A117V^, *Mmd2*^R127P/+^, *Mmd2*^R127P/R127P^, and *Mmd2*^−/−^ mice were significantly higher compared with those in *Mmd2*^+/+^ mice (Fig. 3 C).

**Figure 4.**
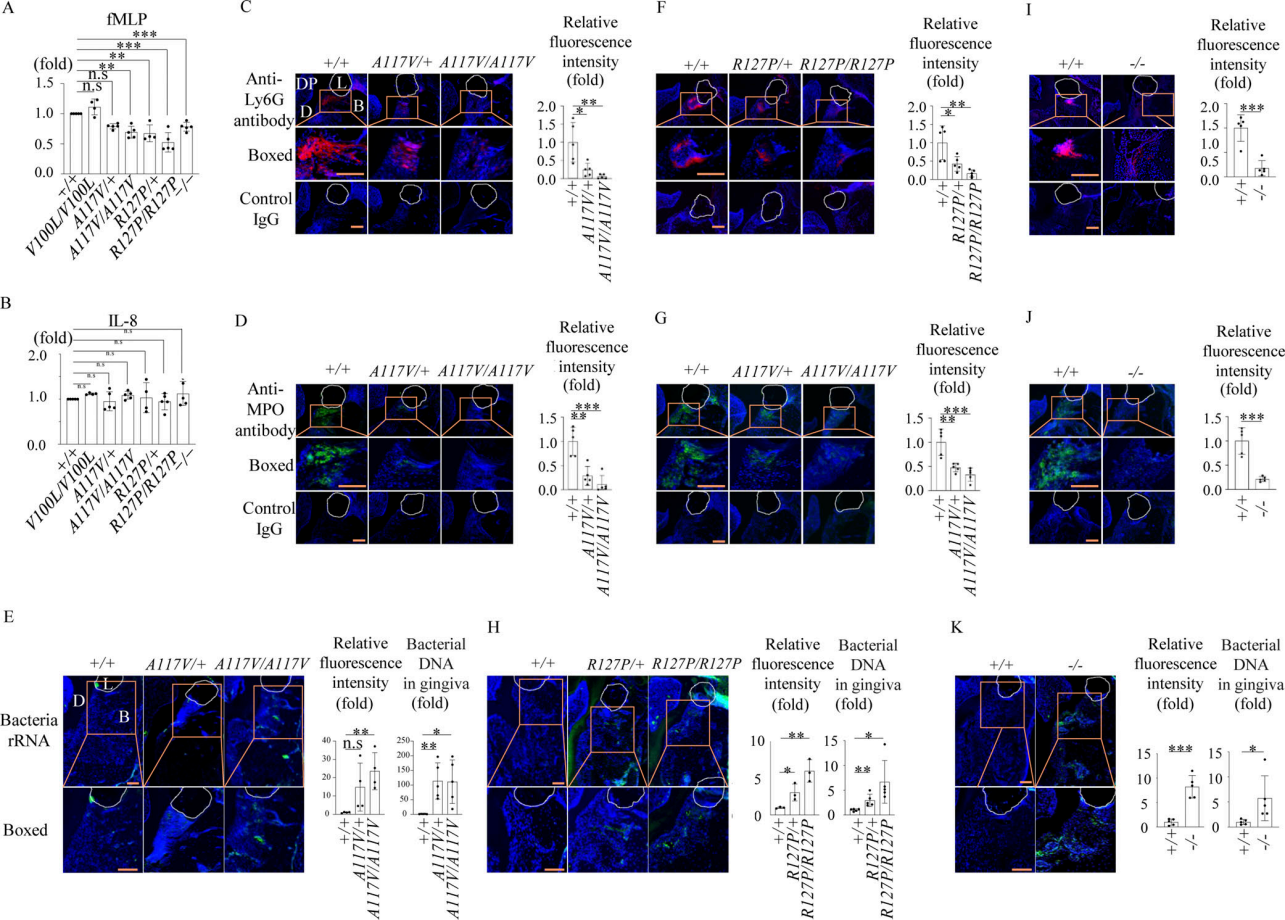
**Neutrophil counts in periodontal tissue in a ligature-induced periodontitis model and neutrophil chemotaxis. (A)** Neutrophil chemotaxis induced by fMLP in *Mmd2*^A117V/+^, *Mmd2*^A117V/A117V^, *Mmd2*^R127P/+^, *Mmd2*^R127P/R127P^, and *Mmd*2^−/−^ mice was decreased compared with that in *Mmd2*^+/+^ and *Mmd2*^V100L/V100L^ mice. (*Mmd2*^+/+^, *n* = 5; *Mmd2*^V100L/V100L^, *n* = 4; *Mmd2*^A117V/+^, *n* = 5; *Mmd2*^A117V/A117V^, *n* = 5; *Mmd2*^R127P/+^, *n* = 4; *Mmd2*^R127P/R127P^, *n* = 5; *Mmd2*^−/−^, *n* = 5). *Mmd2*^+/+^ versus *Mmd2*^V100L/V100L^; P = 0.6758, *Mmd2*^+/+^ versus *Mmd2*^A117V/+^; P = 0.0502, *Mmd2*^+/+^ versus *Mmd2*^A117V/A117V^; P = 0.0015, *Mmd2*^+/+^ versus *Mmd2*^R127P/+^; P = 0.0015, *Mmd2*^+/+^ versus *Mmd2*^R127P/R127P^; P < 0.0001, *Mmd2*^+/+^ versus *Mmd2*^−/−^; P < 0.0001. **(B)** No statistical difference in neutrophil chemotaxis induced by IL-8. The results are expressed as the mean ± SD. (*Mmd2*^+/+^, *n* = 5; *Mmd2*^V100L/V100L^, *n* = 4; *Mmd2*^A117V/+^, *n* = 5; *Mmd2*^A117V/A117V^, *n* = 5; *Mmd2*^R127P/+^, *n* = 4; *Mmd2*^R127P/R127P^, *n* = 5; *Mmd2*^−/−^, *n* = 5). *Mmd2*^+/+^ versus *Mmd2*^V100L/V100L^; P = 0.9669, *Mmd2*^+/+^ versus *Mmd2*^A117V/+^; P = 0.9997, *Mmd2*^+/+^ versus *Mmd2*^A117V/A117V^; P = 0.9913, *Mmd2*^+/+^ versus *Mmd2*^R127P/+^; P > 0.9999, *Mmd2*^+/+^ versus *Mmd2*^R127P/R127P^; P = 0.9994, *Mmd2*^+/+^ versus *Mmd2*^−/−^; P = 0.9440. **(C–K)** Neutrophil numbers were decreased in ligature-induced periodontitis. Immunofluorescence for Ly6G (C, F, and I) and MPO (D, G, and J) in gingival tissues at 24 h after ligation. Red = Ly6G-positive cells, green = MPO, L (white dot line) = ligature, B = alveolar bone, DP = dental pulp, D = dentin. Nuclei were visualized by DAPI (blue). *n* = 5. **(C)***Mmd2*^+/+^ versus *Mmd2*^A117V/+^; P = 0.0185, *Mmd2*^+/+^ versus *Mmd2*^A117V/A117V^; P = 0.0048. **(D)***Mmd2*^+/+^ versus *Mmd2*^A117V/+^; P = 0.0004, *Mmd2*^+/+^ versus *Mmd2*^A117V/A117V^; P = 0.0018. **(E)** Relative fluorescence intensity: *Mmd2*^+/+^ versus *Mmd2*^A117V/+^; P = 0.0790, *Mmd2*^+/+^ versus *Mmd2*^A117V/A117V^; P = 0.0043, bacterial DNA in gingival tissues: *Mmd2*^+/+^ versus *Mmd2*^A117V/+^; P = 0.0036, *Mmd2*^+/+^ versus *Mmd2*^A117V/A117V^; P = 0.0108. **(F)***Mmd2*^+/+^ versus *Mmd2*^R127P/+^; P = 0.0339, *Mmd2*^+/+^ versus *Mmd2*^R127P/R127P^; P = 0.0042. **(G)***Mmd2*^+/+^ versus *Mmd2*^R127P/+^; P = 0.0018, *Mmd2*^+/+^ versus *Mmd2*^R127P/R127P^; P = 0.0002. **(H)** Relative fluorescence intensity: *Mmd2*^+/+^ versus *Mmd2*^R127P/+^; P = 0.0371, *Mmd2*^+/+^ versus *Mmd2*^R127P/R127P^; P = 0.0049, bacterial DNA in gingival tissues: *Mmd2*^+/+^ versus *Mmd2*^R127P/+^; P = 0.0090, *Mmd2*^+/+^ versus *Mmd2*^R127P/R127P^; P = 0.0185. **(I)***Mmd2*^+/+^ versus *Mmd2*^−/−^; P = 0.0004). **(J)***Mmd2*^+/+^ versus *Mmd2*^−/−^; P = 0.0002). **(K)** Relative fluorescence intensity: *Mmd2*^+/+^ versus *Mmd2*^−/−^; P = 0.0001, bacterial DNA in gingival tissues: *Mmd2*^+/+^ versus *Mmd2*^−/−^; P = 0.0466. **(E, H, and K)** Fluorescence in situ hybridization (FISH) for bacteria rRNA in gingival tissues at 24 h after ligation. Quantification for relative fluorescence intensity were analyzed by BZ-X800 Analyzer (Keyence) in a random field. Green = bacteria rRNA. *n* = 4–5. Scale bar = 100 μm. Relative amounts of bacteria 16S rDNA in the gingival tissues analyzed by qPCR. The average of 16S rDNA levels in the gingival tissues from *Mmd2*^+/+^ with ligatures was set as 1. Data are presented as mean ± SD. *P < 0.05, **P < 0.01, and ***P < 0.001 with one-way ANOVA with Tukey–Kramer test (A–H) or two-tailed unpaired *t* test (I–K). n.s = not significant. Each dot represents a biologically independent mouse. All representative images from more than three independent experiments with similar results.

Neutrophil chemotaxis, assessed via fMLP stimulation, was significantly reduced in *Mmd2*^A117V/+^, *Mmd2*^A117V/A117V^, *Mmd2*^R127P/+^, *Mmd2*^R127P/R127P^, and *Mmd2*^−/−^ mice, but was unaffected by IL-8 stimulation (Fig. 4, A and B). We further investigated neutrophil infiltration histologically (Kim et al., 2023). As expected, the periodontal tissues of *Mmd2*^A117V/+^, *Mmd2*^A117V/A117V^, *Mmd2*^R127P/+^, *Mmd2*^R127P/R127P^, and *Mmd2*^−/−^ mice exhibited a reduced number of neutrophils (anti-Ly6G and anti-MPO antibodies positive cells), along with an increased bacterial load compared with *Mmd2*^+/+^ mice in the periodontitis model (Fig. 4, C–K). Taken together, the *Mmd2*^A117V/+^, *Mmd2*^A117V/A117V^, *Mmd2*^R127P/+^, *Mmd2*^R127P/R127P^, and *Mmd2*^−/−^ mice replicated the patient phenotype, characterized by severe alveolar bone loss and defective neutrophil chemotaxis, supporting the pathogenic role of p.A116V and p.R126P mutations in aggressive periodontitis.

### Mutant MMD2 impair fMLP-induced phosphorylation of ERK

**Figure 5.**
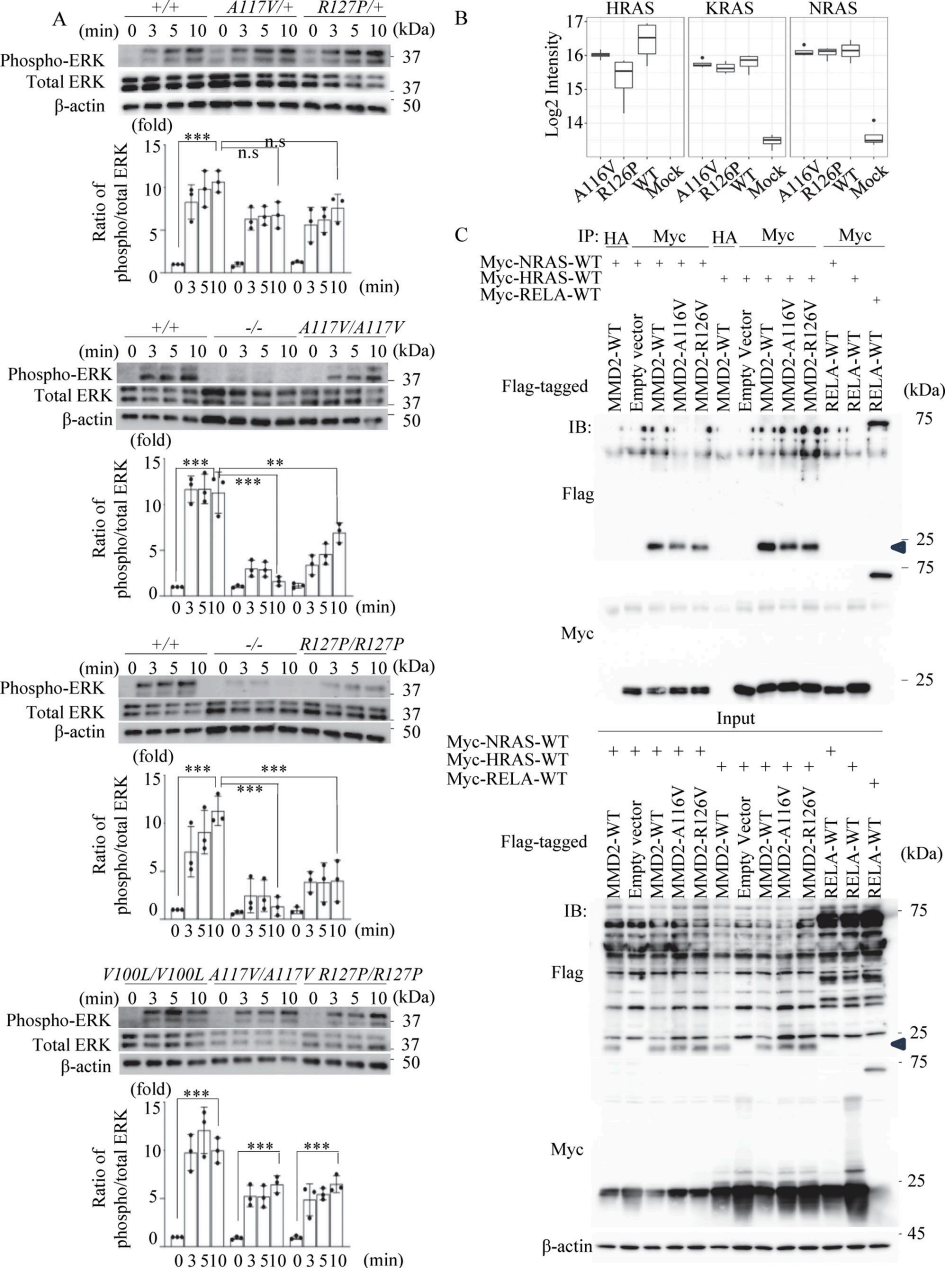
**Phosphorylation of ERK- and DIA-MS–based interaction assay. (A)** The activation of ERK phosphorylation of neutrophils from *Mmd2*^+/+^, *Mmd2*^−/−^, *Mmd2*^A117V/+^, *Mmd2*^A117V/A117V^, *Mmd2*^R127P/+^, *Mmd2*^R127P/R127P^, and *Mmd2*^V100L/V100L^ mice. Neutrophils were stimulated with fMLP for 0, 3, 5, and 10 min, and the cell lysate was used in immunoblotting, using antibodies against phosphorylated ERK and total ERK. The same experiment was carried out at least three times, and one set of representative data is shown. Data are presented as mean ± SD. **P < 0.01 and ***P < 0.001 with one-way ANOVA with Tukey–Kramer test. ns = not significant *n* = 3. 0 min versus 10 min in *Mmd2*^+/+^; P < 0.0001. 10 min in *Mmd2*^+/+^ versus 10 min in *Mmd2*^A117V/+^; P = 0.0816. 10 min in *Mmd2*^+/+^ versus 10 min in *Mmd2*^R127P/+^; P = 0.3123. 0 min versus 10 min in *Mmd2*^+/+^; P < 0.0001. 10 min in *Mmd2*^+/+^ versus 10 min in *Mmd2*^−/−^; P < 0.0001. 10 min in *Mmd2*^+/+^ versus 10 min in *Mmd2*^A117V/A117V^; P = 0.0033. 0 min versus 10 min in *Mmd2*^V100L/V100L^; P < 0.0001. 0 min versus 10 min in *Mmd2*^A117V/A117V^; P = 0.0006. 0 min versus 10 min in *Mmd2*^R127P/R127P^; P = 0.0006. **(B)** Mass spectrometry of interacting Ras proteins after immunoprecipitating MMD2. Binding of RAS to mutated MMD2. **(C)** To examine the interaction between MMD2 and RAS, co-immunoprecipitation analysis was performed by co-expressing Flag-tagged MMD2-WT or MMD2-mut with Myc-tagged NRAS or HRAS. As a control for co-IP, a sample immunoprecipitated with an anti-HA antibody was included. Additionally, co-immunoprecipitation was performed with RelA, which has not been reported to interact with RAS, as a negative control. MMD2-derived band is highlighted with blue arrowhead. Source data are available for this figure: SourceData F5.

Neutrophils from mouse were extracted and subjected to immunoblot to assess phosphorylation of ERK following fMLP stimulation, as ERK signaling is important for neutrophil chemotaxis (Lee et al., 2010; Zhang et al., 2016). Neutrophils from *Mmd2*^A117V/+^, *Mmd2*^A117V/A117V^, *Mmd2*^R127P/+^, and *Mmd2*^R127P/R127P^ mice showed mildly reduced ERK phosphorylation in response to fMLP stimulation compared neutrophils from *Mmd2*^+/+^ mice (Fig. 5 A). Furthermore, the impairment of ERK phosphorylation was pronounced in *Mmd2*^−/−^ mice. Similar to the *Mmd2*^+/+^ mice, neutrophils from *Mmd2*^V100L/V100L^ mice showed stronger ERK phosphorylation than those from *Mmd2*^A117V/A117V^ and *Mmd2*^R127P/R127P^ mice (Fig. 5 A, bottom).

The chemotaxis of neutrophils toward bacterial fMLP is a crucial process in immune defense against bacterial invasion (Wille et al., 2018). In this process, the fMLP activates Ras and Raf, which in turn activates ERK, supporting chemotaxis toward fMLP (Zhang et al., 2016). Ras localizes in the cell membrane and in the Golgi apparatus and regulates cell function through activating ERK (Jin et al., 2012; Peng et al., 2014). MMD2 activates Ras by binding to it, which in turn activates the Ras/ERK signaling pathway in the Golgi apparatus (Jin et al., 2012). The interaction between MMD2 and Ras was examined using Data-Independent Acquisition Mass Spectrometry (DIA-MS) in transient gene expression experiments with HEK293T cells. Although the difference may not be significant, the p.A116V and p.R126P mutant MMD2 exhibited a potential decrease in binding to HRAS compared with WT MMD2 in this assay (Fig. 5 B). On the other hand, no significant difference was observed in the binding affinity of these mutant MMD2 proteins to KRAS and NRAS. The interaction between MMD2 and Ras was also examined in transient gene expression experiments after immunoprecipitating overexpressed MMD2 in HEK293T cells. Consistent with the DIA-MS results, both mutant MMD2 proteins showed a slight decrease in binding to HRAS. However, in contrast to the DIA-MS results, both mutants also exhibited impaired binding to NRAS (Fig. 5 C and Fig. S3 H). Although more detailed and precise studies are needed to confirm these observations, these findings suggest that mutant MMD2 may alter its binding to Ras.

### Concluding remarks

We reported the first Mendelian disease associated with monoallelic mutations in *MMD2* causing the autosomal dominant form of aggressive periodontitis. Previous studies of monogenic diseases have revealed that the lack of functional neutrophils in the periodontal tissues causes severe periodontitis. Patients with p.A116V or p.R126P mutations in *MMD2*, similar to patients with PLS and CHS, showed impaired chemotaxis triggered by fMLP stimulation. Patients with heterozygous p.A116V in *MMD2* showed mildly decreased numbers of total leukocytes, but their absolute neutrophil numbers were not strongly affected. The knock-in mouse models, *Mmd2*^A117V/+^, *Mmd2*^A117V/A117V^, *Mmd2*^R127P/+^, and *Mmd2*^R127P/R127P^, along with *Mmd2*^−/−^, presented alveolar bone loss in a ligature-induced periodontitis model. Additionally, neutrophils from these knock-in and knockout mice showed impairment of chemotaxis. These results suggest that the knock-in mouse with the *MMD2* mutations identified in patients successfully replicated the patients’ phenotype, supporting the disease concept of a Mendelian disease due to monoallelic mutations in *MMD2*.

In mice, Mmd2 plays crucial migratory and metabolic roles during astrogliogenesis (Kang et al., 2012). However, due to the absence of MMD2-deficient patients, the function of MMD2 in humans remains incompletely understood. In the current study, abnormalities of Golgi apparatus proteins were pointed out in patients’ neutrophil. The Golgi apparatus has recently been recognized as a crucial contributor to innate immune signaling pathways (Mărunţelu et al., 2024). For example, in patients with LAD2 deficiency, the loss of Golgi fucosylation due to a defect in the *SLC35C1* gene, which encodes a GDP-fucose transporter, results in the absence of sialyl Lewis X and other structurally related fucosylated selectin ligands, causing a rolling defect in neutrophils (Hanna and Etzioni, 2012). Intriguingly, MMD2 is exclusively localized in the Golgi apparatus, and the p.A116V and p.R126P mutation in *MMD2* did not alter its mRNA and protein expression. Therefore, the expression of mutant MMD2 protein may relate to the abnormalities in Golgi apparatus proteins, impacting neutrophil functions (Hanna and Etzioni, 2012). Additionally, as in patients with p.A116V or p.R126P mutation, neutrophils from the knock-in and knockout mice displayed impaired fMLP-induced chemotaxis. Decreased ERK activation, as assessed by fMLP-induced ERK phosphorylation, was observed in neutrophils from *Mmd2*^−/−^, *Mmd2*^A117V/A117V^, and *Mmd2*^R127P/R127P^ mice. Furthermore, the presence of impairment in interaction between mutant MMD2 and HRAS was identified by DIA-MS–based interaction studies using transient expression systems. These results suggest that monoallelic mutations in *MMD2* may affect Golgi apparatus proteins and Ras-dependent MAPK pathway, leading to the functional defects in neutrophils.

In summary, our study highlights that impairment of neutrophil chemotaxis could be caused by functional impairment of MMD2, contributing to the molecular pathogenesis of aggressive periodontitis. It also indicates that, in some patients with aggressive periodontitis, there is a genetic cause behind the condition. Further accumulation of cases will be required to obtain a precise understanding of aggressive periodontitis associated with monoallelic mutations in *MMD2*.

## Materials and methods

### Study families

#### Family A

All affected subjects (A-II-5, A-III-2, A-III-3, A-III-4, A-IV-1, and A-IV-3) were diagnosed with aggressive periodontitis according to periodontal and X-ray examinations. Blood was collected from these four affected subjects and five unaffected subjects (A-II-1, 2, 4, 6, and A-III-5) in this family for genetic analyses. Later blood was collected from A-IV-1, 2, 3, 4, and 5. Additionally, blood and bone marrow samples were collected from patients A-III-2 and A-III-4, and FACS analysis, chemotaxis assay, and computed tomography imaging were performed.

#### Family B

Patients B-II-2 and B-III-1 were diagnosed with aggressive periodontitis based on periodontal and X-ray examinations. Blood was collected from patient B-III-1 for genetic analysis and chemotaxis assay. However, at present, consent for blood collection has not been obtained from patient B-II-2.

### Exome sequencing and variant filtering

The gDNA libraries were prepared using a SeqCap EZ Human Exome Library v2.0 (Roche). Sequencing was performed with 100-bp paired-end reads using the HiSeq2000 sequencer (Illumina). We used Burrows–Wheeler aligner for alignment and mapping and SAMtools (RRID:SCR_002105) and Picard for SAM/BAM. Exome sequencing was performed using the GATK and SAMtools for variant calls and Annovar for annotation. Functional predictions of amino acid changes were performed using PolyPhen-2, Mutation Taster, SIFT, and CADD. Control exome sequences were obtained from Japanese patients undergoing exome sequencing analysis for other diseases. All reported genomic coordinates were in GRCh37/hg19. The identified mutation was confirmed by standard PCR-based amplification, followed by sequence analysis using Applied Biosystems 3130 DNA sequencer (Thermo Fisher Scientific). The primer sequences and PCR conditions are available upon request.

### Linkage analysis

The samples used for linkage analysis were A-II-1, A-II-5, A-II-6, A-III-2, A-III-3, A-III-4, and A-III-5. gDNA was extracted from the venous blood of each subject in accordance with standard protocols. We used the Genome-Wide Human SNP Array 6.0 (Affymetrix) for genotyping single-nucleotide polymorphisms and performed linkage analysis using Allegro software, assuming dominant inheritance.

### Long-read sequencing

Library preparation was conducted utilizing a ligation sequencing kit (SQK-LSK-109; Oxford Nanopore Technologies) following the manufacturer’s protocol. Sequencing was performed using the MinION device and R9.4.1 flow cells (Oxford Nanopore Technologies). Guppy (v.3.2.2, https://nanoporetech.com/software/other/guppy) was used for base calling, and LAST (v.983, https://github.com/mcfrith/last-genome-alignments) was used for mapping.

### In silico modeling

3D experimental structures were retrieved from the Protein Data Bank (Research Collaboratory for Structural Bioinformatics Protein Data Bank (RCSB PDB), RRID:SCR_012820). Since the structure of human MMD2 protein has not been determined, we created a homology model based on the structure of the human adiponectin receptor 1 protein (PDB code: 5LXG) using MOE software (Molecular Operating Environment 2013.08; Chemical Computing Group Inc., 2013. https://www.chemcomp.com). The structural figures were prepared with PyMOL (RRID:SCR_000305) 2.5 (https://pymol.org).

### ROS assay

Dihydrorhodamine 123 (f.c. 100 mM, cat-no P8139; Sigma-Aldrich) was added to 100,000 neutrophils suspended in Hank’s balanced salt solution. After 5 min incubation in a 37°C shaking water bath, the cells were transferred to tubes with or without PMA (f.c. 2 mg/ml, cat-no D1054; Sigma-Aldrich), opsonized zymosan (f.c. 200 mg/ml), heat-killed *Escherichia coli* (1 × 10^8^ cells), and heat-killed *Staphylococcus aureus* (1 × 10^8^ cells). Each tube was incubated in a 37°C shaking water bath for an additional 30 min (for PMA and zymosan) or 1 h (for *E. coli* and *S. aureus*) before analysis by flow cytometry. Following incubation, samples were immediately analyzed by flow cytometry using the BD FACSVerse (BD Biosciences) and FlowJo (RRID:SCR_008520) software (Tree Star Software).

### Analysis of CD34-positive cells in peripheral blood

Mononuclear cells were isolated from peripheral blood using Lymphoprep (cat-no 07801; STEMCELL Technologies), followed by the lysis of RBCs. Mononuclear cells were then incubated with FITC Anti-Human CD45 antibody (cat-no 555482; BD Pharmingen), PE Anti-HumanCD34 PE antibody (cat-no 555822; BD Pharmingen), and anti APC Anti-Human CD14 antibody (cat-no 555399; BD Pharmingen) in PBS with 2% FBS for 15 min at dark. The cells were then washed with PBS twice and resuspended in PBS with 2% FBS. Samples were immediately analyzed by flow cytometry using the BD FACSVerse and FlowJo software.

### Immunohistochemical staining

Some sections were stained with anti-CD34 (1:200, ab81289; Abcam). Following sequential incubation with goat anti-rabbit IgG-HRP (R&D Systems) (1:300), the sections were treated with a diaminobenzidine solution (DAKO) to visualize the immunoreactivity.

### CFU assay

Bone marrow CD34-positive cells were magnetically separated using CD34 MicroBead Kit, UltraPure (cat#130-100-453; Miltenyi Biotec), in accordance with the manufacturer’s instructions. One thousand CD34-positive cells were mixed in methocult (cat#H4230; STEMCELL Technologies) with recombinant human SCF (50 ng/ml, cat#255-SC-050; R&D Systems), recombinant human IL-3 (50 ng/ml, cat#203-IL-050; R&D Systems), recombinant human thrombopoietin (5 ng/ml, cat#288-TP-025; R&D Systems), and recombinant human G-CSF (50 ng/ml, cat#214-CS-025; R&D Systems) and plated in a 35-mm dish. The numbers and distribution of colonies were assessed on day 12 after seeding.

### Liquid suspension culture

Bone marrow CD34-positive cells were magnetically separated using CD34 MicroBead Kit, UltraPure, and 20,000 CD34-positive cells were cultured in StemPro-34 SFM medium (cat#10639011; Thermo Fisher Scientific) supplemented with recombinant human IL-3 (100 ng/ml), recombinant human stem cell factor (100 ng/ml), and recombinant human G-CSF (100 ng/ml). On day 16, the cells were stained with fluorescein isothiocyanate anti-human CD15 (cat#562370; BD Biosciences), APC anti-human CD33 (cat#551378; BD Biosciences), and APC-Cy7 anti-human CD11b (cat#557873; BioLegend) and analyzed using FACSVerse (BD Biosciences).

### Generation of mouse model using platinum TALEN and CRISPR-Cas9 gene editing

Mice carrying the *Mmd2* A117V, R127P, and V100L variant were generated using the TALEN and CRISPR-Cas9 gene editing tool. The TALEN pair that showed high targeting efficiency and low off-target effects was used for in vitro transcription with the MEGAscript T7 Transcription Kit (Thermo Fisher Scientific). TALEN mRNAs were combined with the ssODN construct and injected into pronuclei of C57BL/6 single-cell mouse embryos. crRNA that showed high targeting efficiency and low off-target effects was constructed in Integrated DNA Technologies. crRMA/TracrRNA, Cas9, and SSODN were injected into pronuclei of C57BL/6 single-cell mouse embryos. We then backcrossed *Mmd2*^A117V/A117V^, *Mmd2*^R127P/R127P^, *Mmd*2^V100L/V100L^, and *Mmd*2^−/−^ mice with C57BL/6 mice for eight generations.

### Immunoblotting

Protein samples were subjected to electrophoresis on a 10% SDS-polyacrylamide gel and transferred to a polyvinylidene difluoride membrane (Bio-Rad Laboratories). After being blocked for 1 h (5% nonfat milk in TBS plus 0.1% Tween 20), the membrane was incubated at 4°C overnight with rabbit anti-mouse pERK (Cell Signaling Technology [CST]), rabbit anti-mouse ERK (CST), rabbit anti-mouse β-actin (CST), rabbit anti-human MMD2 (CUSABIO Technology), and mouse anti-human β-actin (CST). The membrane was washed twice and incubated with HRP-conjugated secondary goat anti-rabbit antibodies (CST) and HRP-conjugated secondary goat anti-mouse antibodies (CST) at room temperature for 1 h. Immunodetection was performed in accordance with the protocols supplied with ECL Prime Western blotting detection reagents (RRID:SCR_008426; Bio-Rad Laboratories).

### Chemotaxis assay

Neutrophils were suspended in RPMI1640 (Nacalai Tesque) with 1,000 mg/liter glucose and penicillin/streptomycin. Chemotaxis was induced with fMLP (100 nM) and IL-8 (50 ng/ml) for 120 min at 37°C and measured using the Boyden chamber method with a 96-well micro-chemotaxis chamber containing a 3-µm pore-sized filter (Cell Biolabs, Inc.).

### DIA-MS–based proteomics

Proteins were isolated from samples containing TRIzol, and DIA-MS was performed following the procedure established by Kawashima et al. (2019), Kawashima et al. (2022). Briefly, chloroform was utilized to phase separate the TRIzol solution, thereby removing the aqueous phase, after which the proteins were precipitated using ethanol. Following the removal of the supernatant, 100 mM Tris (pH 8.0) containing 4% SDS and 20 mM NaCl was added, and the proteins were solubilized using a Bioruptor II (CosmoBio). The proteins were subsequently digested with Trypsin Platinum (Promega), and the peptide concentration was adjusted to 100 ng/μl using a Fluorometric Peptide Assay (Thermo Fisher Scientific). For liquid chromatography (LC) separation, the peptides were injected into an UltiMate 3000 RSLCnano LC system (Thermo Fisher Scientific). The mass spectrometric peptide spectra were detected using an Orbitrap Exploris 480 mass spectrometer (Thermo Fisher Scientific). Finally, the LC-MS/MS raw data were searched against human spectral libraries, and the proteins and peptides were identified using DIA-NN (version 1.8.1) (Demichev et al., 2020). Quantitative values were calculated for proteins for which both precursor false discovery rate (FDR) and protein FDR were below 1%.

### Proteomic data analysis

Proteomic data were analyzed using R (version 4.3) and Bioconductor (version 3.18) packages. UMAP was conducted using the umap R package (https://github.com/tkonopka/umap) and visualized with the ggplot2 R package (https://ggplot2.tidyverse.org). The DEP R package was employed to perform DEA and hierarchical clustering analysis (Zhang et al., 2017). For the DEA, an absolute log-fold change >1.5 and an adjusted P value of <0.05, when comparing patients to healthy controls, were considered statistically significant. In the hierarchical clustering analysis, the similarities between patients and healthy controls were assessed based on the results of the DEA. GSEA was performed using the clusterProfiler package (Yu et al., 2012). In the GSEA, all proteomic data were ranked in descending order of log-fold change, and GO terms related to biological processes were utilized for functional analysis. The statistical significance threshold was set at a P value of <0.05. GO analysis of the proteins identified through DEA was conducted using the online platform Metascape with the statistical significance threshold of a P value set to <0.05 (Zhou et al., 2019).

### Ligature-induced periodontitis mouse model

Periodontal inflammation and bone loss in a ligature-induced periodontitis model were initiated by the abundant local accumulation of bacteria on ligated molar teeth (Abe and Hajishengallis, 2013). To this end, a 5-0 silk ligature was tied around the maxillary second molar in 8-week-old male and female mice for 7 days or 24 h. After jaws were cleared of remaining tissue, they were kept in 10% hydrogen peroxide overnight. The distance between the cement–enamel junction and alveolar bone crest was examined.

### RNA isolation

RNA was extracted by TriZol RNA extraction reagent (Takara). Maxillary gingival tissues at the palatal side (1 × 3 mm) was used for RNA extraction. Gingival tissues were homogenized by a tissue grinder.

### Reverse transcription-quantitative PCR analysis

Total RNA was isolated with RNAiso Plus (Takara), and 1 µg of RNA was reverse transcribed using the High-Capacity cDNA reverse transcription kit (Thermo Fisher Scientific). quantitative PCR (qPCR) was performed using PowerUp SYBR Green master mix (Thermo Fisher Scientific) and analyzed with the StepOne and StepOnePlus Real-Time PCR Systems (Thermo Fisher Scientific). Relative gene expression levels were calculated using a relative-standard curve method. Each gene expression levels were normalized by the expression levels of 18S, which was used for normalization. 18S; forward (5′ to 3′): 5′-AAG​TTC​CAG​CAC​ATT​TTG​CGA​GTA-3′ and reverse (5′ to 3′): 5′-TTG​GTG​AGG​TCG​ATG​TCT​GCT​TTC-3′, Il6; forward (5′ to 3′): 5′-AAC​GAT​GAT​GCA​CTT​GCA​GA-3′ and reverse (5′ to 3′): 5′-CCA​GAG​GAA​ATT​TTC​AAT​AGG​C-3′, Il1β; forward (5′ to 3′): 5′-AGT​TGA​CGG​ACC​CCA​AAA​G-3′ and reverse (5′ to 3′): 5′-AGC​TGG​ATG​CTC​TCA​TCA​GG-3′, and Ccl3; forward (5′ to 3′): 5′-ACA​CTC​TGC​AAC​CAA​GTC​TTC-3′ and reverse (5′ to 3'): 5′-CTG​CCG​GTT​TCT​CTT​AGT​CAG​G-3′.

### Histology and immunofluorescent staining

The maxillae of the mice were fixed in 4% paraformaldehyde in PBS at 4°C. The maxillae were decalcified in EDTA (0.5 M, pH = 7.2) and then embedded with paraffin. Tissues were sectioned in the coronal plane at a thickness of 6 μm and subjected to immunofluorescent staining. Deparaffinized paraffin sections were rehydrated and incubated with L.A.B. Solution (Liberate Antibody Binding Solution, Polysciences, Inc.) to retrieve the epitope for 5 min at room temperature, followed by treatment with 3% H_2_O_2_ for 10 min. After blocking with Blocking One Histo (Nacalai Tesque, Inc.) for 5 min, the sections were incubated with anti-neutrophil antibody (anti-Ly6G, 551459; BD Pharmingen), anti-Myeloperoxidase antibody (anti-MPO, ab65871; abcam), or control IgG at 4°C for overnight, followed by incubation with secondary antibody (Goat Anti-Mouse IgG H&L [Alexa Fluor 594] [ab150116] or Goat Anti-Rabbit IgG H&L [Alexa Fluor 488] [ab150077]) for 1 h at room temperature. Nuclei were stained with DAPI. Fluorescent images were acquired with BZ-X800 microscope (Keyence). Quantification for relative fluorescence intensity were analyzed by BZ-X800 Analyzer software (Keyence) in a random field and performed by personnel blinded to sample information.

### Fluorescence in situ hybridization

The maxillae of the mice were fixed in 4% paraformaldehyde in PBS at 4°C. They were decalcified in EDTA (0.5 M, pH = 7.2) and then embedded in paraffin. The tissues were sectioned in the coronal plane at a thickness of 6 μm and subjected to fluorescence in situ hybridization. After deparaffinization, the sections were rehydrated and treated with proteinase K (10 μg/ml, Norgen Biotek Corp.) at 37°C for 5 min, followed by rinsing with PBS three times for 5 min each. Then, the sections were immersed in 1 × G-Wash (Genostaff) and hybridized with the Alexa Fluor 488–labelled probe EUB338 (5′–GCT​GCC​TCC​CGT​AGG​AGT-3′) at 46°C for 2 h. After hybridization, the sections were immersed in wash buffer (30% formamide, 0.9 M NaCl, 0.01% SDS, and 20 mM Tris-HCl, pH 7.2) at 46°C for 10 min and then rinsed with PBS. Nuclei were stained with DAPI. Fluorescent images were acquired and analyzed with BZ-X800 microscope (Keyence).

### qPCR analysis of bacterial DNA in gingival tissues

DNAs within tissues were isolated from the gingival tissues (1-mm width) at the palatal side and jawbone. To remove bacteria on the tissue surface, tissues were washed with following procedures: Gingival tissues were sonicated in PBS for 1 min and then vortexed for 10 s. The washing step was repeated three times by replacing PBS. Gingival tissues were digested with 100 μg/ml proteinase K solution (in 100 mM Tris, 5 mM EDTA, 200 mM NaCl, and 0.2% SDS), and DNA was isolated by ethanol precipitation. Tissue DNAs were subjected to qPCR using a primer set targeting bacterial 16S rDNA (337F and 907R, forward [5′ to 3′]: 5′-GACTCCTACGGGAGGCWGCAG-3′ [337F] and reverse (5′ to 3′): 5′-CCGTCAATTCCTTTRAGTTT-3′ [907R]) to assess the bacterial load in tissues. 16S rDNA levels were normalized by mouse genomic DNA amplified by primers targeting the mouse 18S gene.

### Sample preparation for proteomics analyses

We obtained the pCMV6 vector encoding human Flag-tagged MMD2 from OriGene Technologies Inc. The MMD2 construct carrying the mutant allele was generated via site-directed mutagenesis using the PfuUltra II Fusion HS DNA Polymerase (Agilent Technologies). HEK293T cells were seeded in 150-mm dish (8 × 10^6^ cells/dish) and transfected with 30 μg of FLAG-tagged MMD2/pCMV6, carrying either WT or each variant, using Lipofectamine LTX (Thermo Fisher Scientific) according to the manufacture’s protocol. 24 h after transfection, each cell pellet was lysed by adding 1 ml of cold lysis buffer (50 mM Tris-HCl, pH 7.5, 150 mM NaCl, 1 mM EDTA, and 0.5% NP40), followed by rotation at 4°C for 30 min. The lysates were centrifuged at 3,000 *g* for 20 min at 4°C to remove debris. The cleared lysates were incubated with 40 μl of the washed anti-FLAG M2 beads at 4°C with rotation for 18 h. After incubation, the beads were washed three times with 1 ml of IP buffer (50 mM Tris-HCl, pH 7.5, 150 mM NaCl, 1 mM EDTA, and 0.05% NP40), followed by a single wash with 1 ml of IP buffer without detergent. At this final step, all residual buffer was carefully removed. Beads were incubated in a buffer containing 2 M urea, 50 mM Tris-HCl, pH 8.0, and 1 mM DTT at 37°C for 30 min. Iodoacetamide was added to a final concentration of 3 mM, and samples were incubated at room temperature for 45 min in the dark. DTT was added to a final concentration of 3 mM and 750 ng of trypsin (trypsin gold, Promega) was added to each sample, followed by overnight incubation at 37°C with shaking. The supernatant was transferred to a fresh tube, and an additional 500 ng trypsin added to the supernatant, followed by incubation for 2 h at 37°C. Digested samples were desalted on BioPureSPN Mini C18 SPE columns (Nest Group) according to the manufacturer’s protocol. Samples were dried by vacuum centrifugation and resuspended in 0.1% formic acid (FA) for mass spectrometry analysis.

### Mass spectrometry data acquisition

IP samples for four replicates were used. All samples were analyzed on an Orbitrap Eclipse mass spectrometry system equipped with an Easy nLC 1,200 ultra-high pressure LC system interfaced via a Nanospray Flex nanoelectrospray source (Thermo Fisher Scientific). Samples were injected onto a fritted fused silica capillary (30 cm × 75-μm inner diameter with a 15-μm tip, CoAnn Technologies) packed with ReprosilPur C18-AQ 1.9-μm particles (Dr. Maisch GmbH). Buffer A consisted of 0.1% FA, and buffer B consisted of 0.1% FA/80% acetonitrile. Peptides were separated by an organic gradient from 5 to 35% mobile buffer B over 120 min, followed by an increase to 100% B over 10 min at a flow rate of 300 nl/min. Analytical columns were equilibrated with 3 μl of buffer A. To build a spectral library, samples from each set of biological replicates were pooled and acquired in data-dependent manner. Data-dependent acquisition (DDA) was performed by acquiring a full scan over a m/z range of 375–1,025 in the Orbitrap at 120,000 resolving power (@ 200 m/z) with a normalized AGC target of 100%, an RF lens setting of 30%, and an instrument-controlled ion injection time. Dynamic exclusion was set to 30 s, with a 10 p.p.m. exclusion width setting. Peptides with charge states 2–6 were selected for MS/MS interrogation using higher energy collisional dissociation (HCD) with a normalized HCD collision energy of 28%, with 3 s of MS/MS scans per cycle. DIA was performed on all subject samples. A full scan was collected at 60,000 resolving power over a scan range of 390–1,010 m/z, an instrument controlled AGC target, an RF lens setting of 30%, and an instrument controlled maximum injection time, followed by DIA scans using 8 m/z isolation windows over 400–1,000 m/z at a normalized HCD collision energy of 28%.

### Mass spectrometry data analysis

The Spectronaut algorithm was used to build spectral libraries from DDA data, identify peptides/proteins, localize phosphorylation sites, and extract intensity information from DIA data (Bruderer et al., 2017). DDA data were searched against the *Homo sapiens* reference proteome sequences in the UniProt database (one protein sequence per gene, downloaded on August 23, 2023). FDRs were estimated using a decoy database strategy (Elias and Gygi, 2007). All data were filtered to achieve a FDR of 0.01 for peptide-spectrum matches, peptide identifications, and protein identifications. Search parameters included a fixed modification for carbamidomethyl cysteine and variable modifications for N-terminal protein acetylation, and methionine oxidation. All other search parameters were Biognosys factory defaults. Statistical analysis of proteomics data was conducted utilizing the MSstats package in R (Choi et al., 2014). Protein interactions were scored with the SAINTq algorithm (Fontaine et al., 2018).

### Binding of MMD2 to RAS

6.8 × 10^6^ HEK293T cells were seeded in a 6-well plate. Plasmids containing either a Myc tag or a Flag tag were transfected at 3 μg each using Lipofectamine LTX. After 30 h, transfected cells were lysed, and a portion of the lysate was collected as a whole lysate to confirm protein expression. The remaining lysate was incubated with 3.5 μg of either an anti-Myc antibody (C3956; Sigma-Aldrich) or an anti-HA antibody (H6908; Sigma-Aldrich) for 16 hours. 250 μg of protein A/G magnetic beads was added and incubated at room temperature for 1 h. The samples were then washed three times, and co-immunoprecipitated samples were collected. The co-immunoprecipitation procedure was carried out according to the protocol of the Pierce Classic Magnetic IP/Co-IP Kit (Thermo Fisher Scientific). Since the eluted antibody bands can mask the target antigen, western blot detection using an anti-Myc antibody was performed with the Thermo Fisher Scientific Clean-Blot IP Detection Reagent (Thermo Fisher Scientific).

### Statistical analysis

All statistical analyses were performed using JMP Pro software, ver.17 (1989–2023; SAS Institute Inc.) One-way ANOVA followed by Dunnett’s multiple comparisons test and the two-tailed unpaired Student’s *t* test were used to determine statistical significance between the control and experimental groups. P values ≤0.05 were considered statistically significant.

### Online supplemental material

Fig. S1 shows the clinical photographs in patients with *MMD2* mutations, population genetics for MMD2, and neutrophil analysis in patients with p.A116V *MMD2* mutation. Fig. S2 shows the characteristics of the patients’ bone marrow and peripheral blood. Fig. S3 shows the generation of *Mmd2* A117V, R127P, and V100L knock-in mice using platinum TALEN and CRISPR-Cas9 and immunophenotyping of patients with p.A116V *MMD2* mutation.

## Supplementary Material

SourceData F2 is the source file for Fig. 2.

SourceData F5 is the source file for Fig. 5.

SourceData FS3 is the source file for Fig. S3.

## Data Availability

All data are available in the article itself and its supplementary materials and are also available upon reasonable request from the corresponding authors.
