# Supplementary figures and images for: Monoallelic mutations in MMD2 cause autosomal dominant aggressive periodontitis
Source: J Exp Med. 2025 Jul 15;222(9):e20231911. doi: 10.1084/jem.20231911 (PMC12262042; doi:10.1084/jem.20231911)

Figure 2B

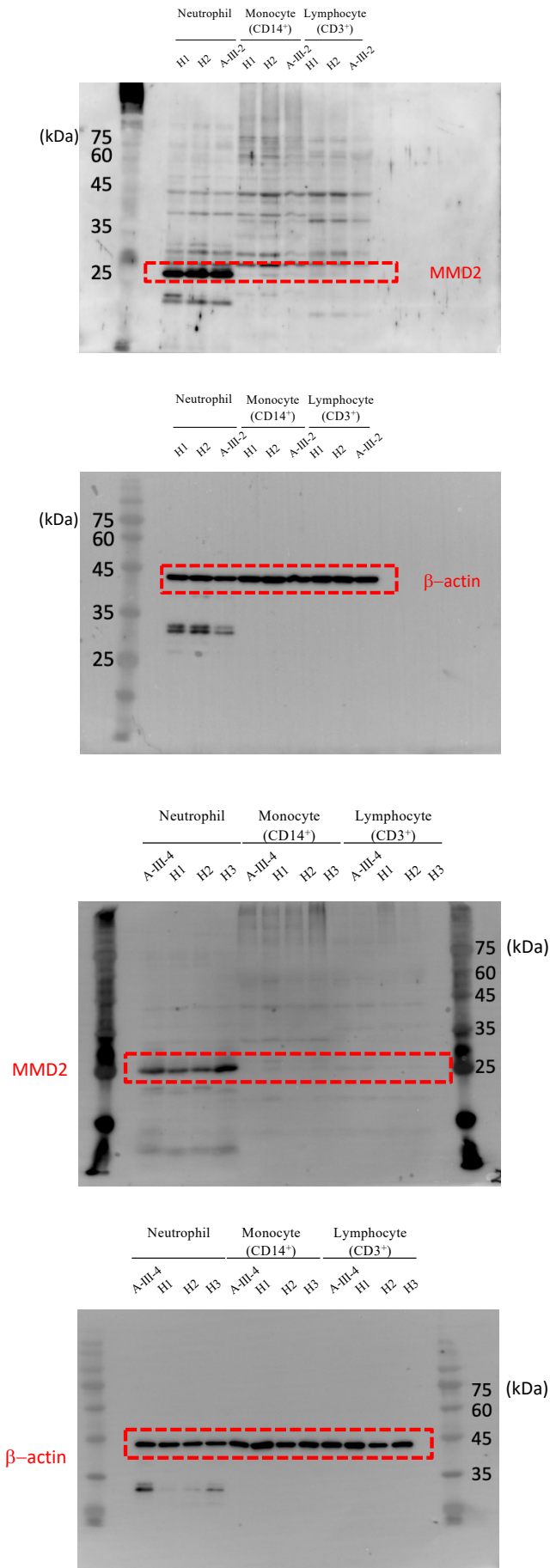

Red boxes: correspond to the cropped blots.

Supplement: SourceData F2 — is the source file for Fig. 2. [file jem_20231911_sourcedataf2.pdf]

Figure S3

D

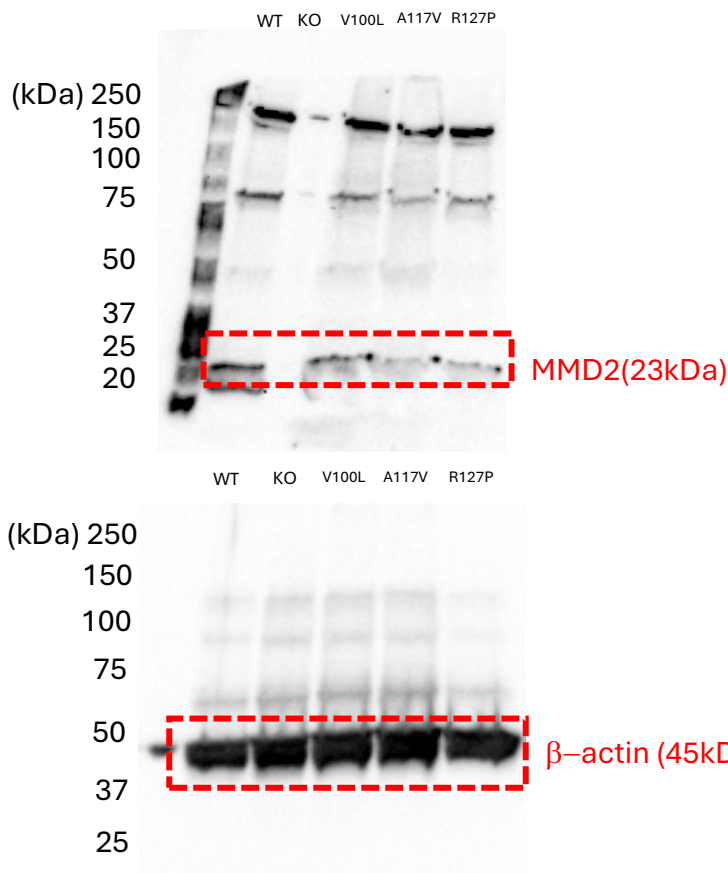

E

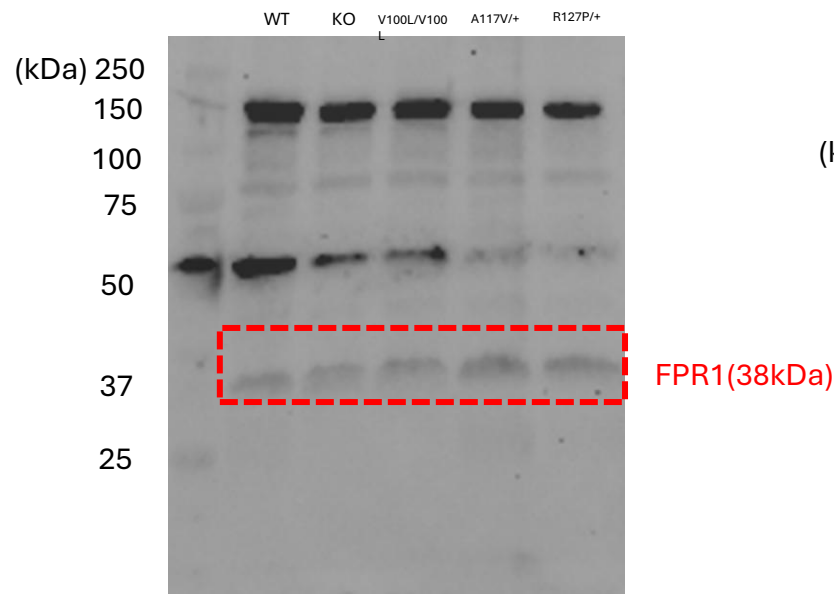

H

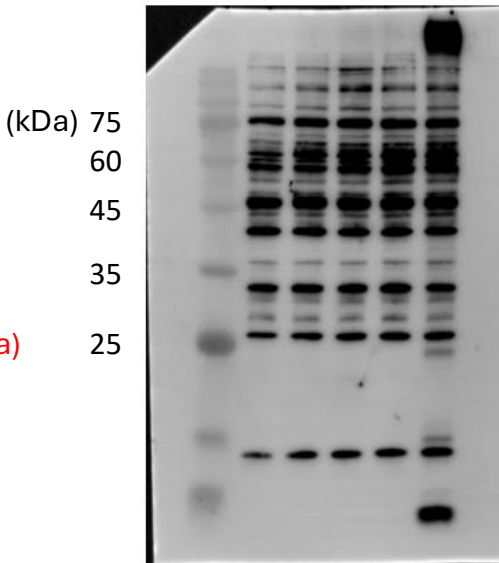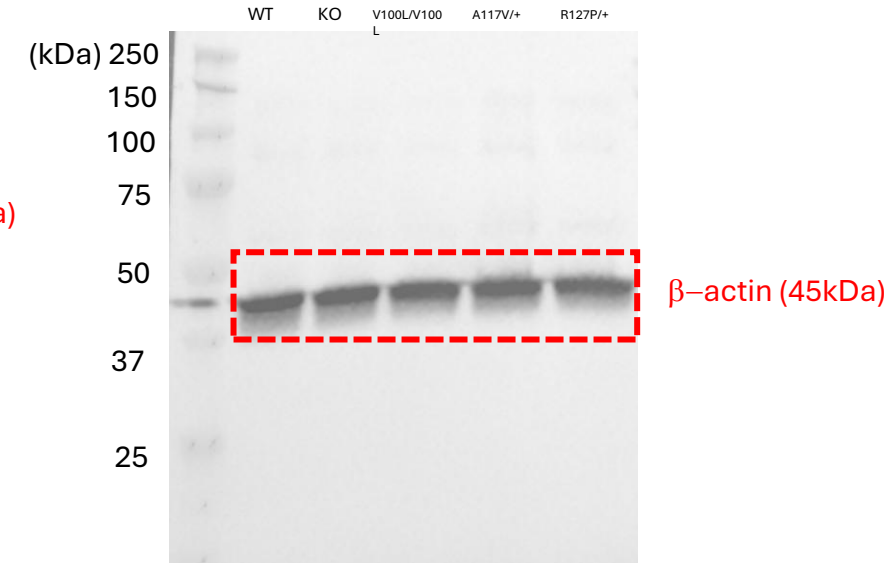

Red boxes: correspond to the cropped blots.

Supplement: SourceData FS3 — is the source file for Fig. S3. [file jem_20231911_sourcedatafs3.pdf]
