# Supplementary material for: Monoallelic mutations in MMD2 cause autosomal dominant aggressive periodontitis
Source: J Exp Med. 2025 Jul 15;222(9):e20231911. doi: 10.1084/jem.20231911 (PMC12262042; doi:10.1084/jem.20231911)
Supplement: SourceData F5 — is the source file for Fig. 5. [file jem_20231911_sourcedataf5.pdf]

Figure 5

A

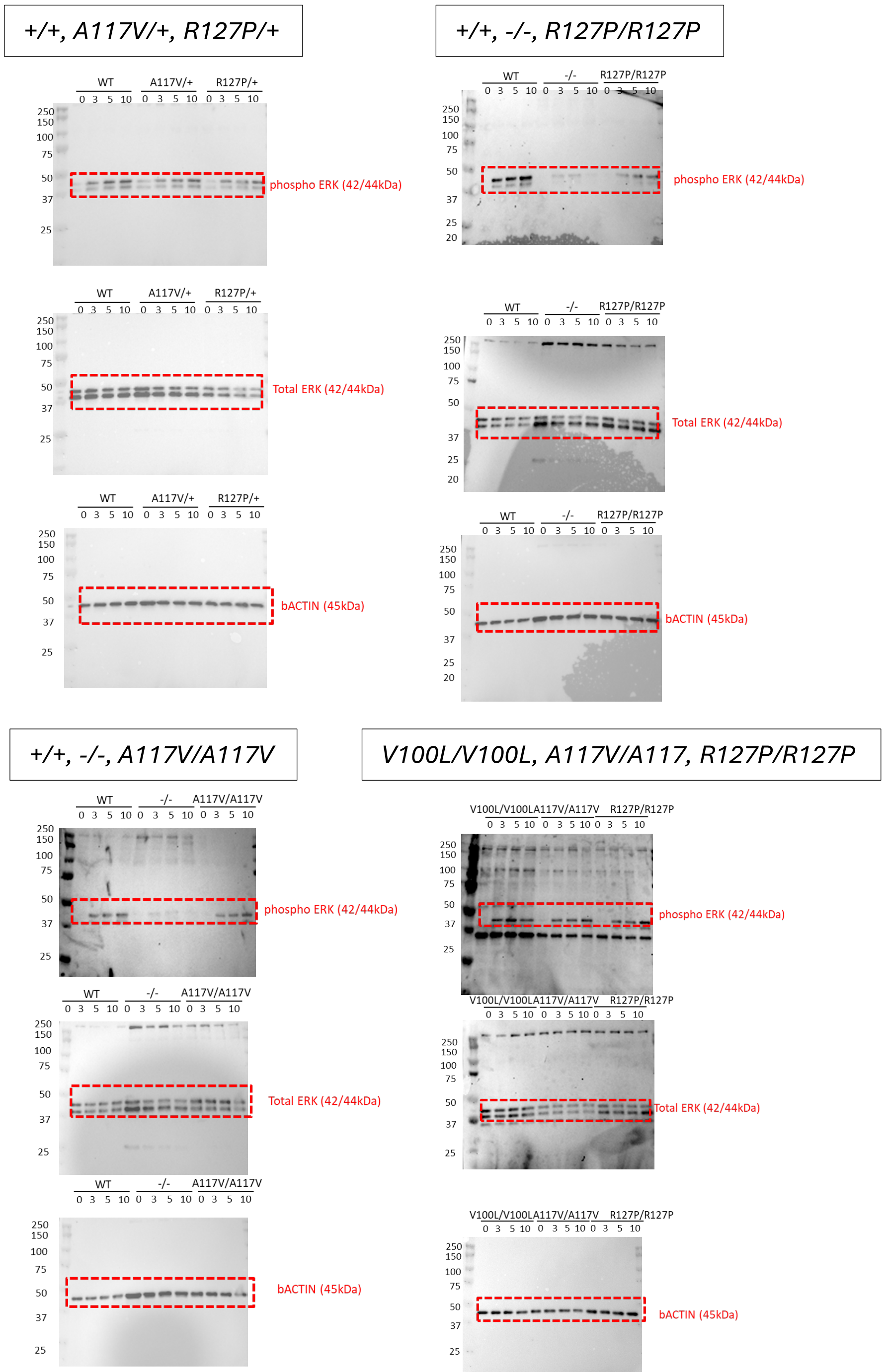

C

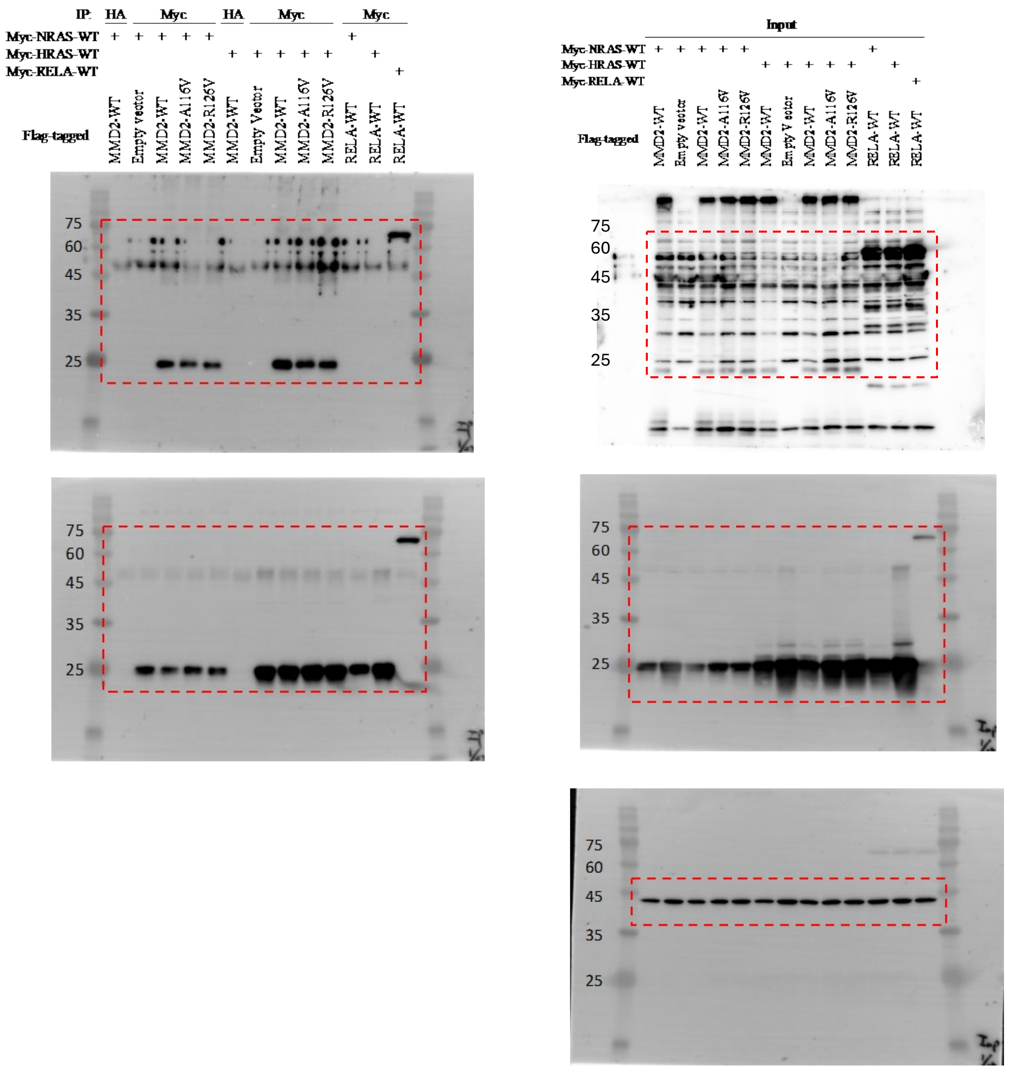

Red boxes: correspond to the cropped blots.
